# Myeloid‐Derived Growth Factor Alleviates Diabetic Non‐Alcoholic Fatty Liver Disease by Up‐Regulating ABCA1 and ABCG1 Expression via the AMPK/PPARγ/LXRα Pathway

**DOI:** 10.1111/jcmm.71428

**Published:** 2026-11-01

**Authors:** Zeyu Chen, Biying Meng, Bingyi Wang, Yan Ding

**Affiliations:** ^1^ School of Medical Informatics and Engineering Hunan Medical College Huaihua Hunan Province China; ^2^ Department of Endocrinology General Hospital of Central Theater Command Wuhan Hubei Province China

**Keywords:** diabetes mellitus, macrophages, myeloid‐derived growth factor, non‐alcoholic fatty liver disease

## Abstract

The regulatory role of bone marrow in systemic metabolic processes remains incompletely characterized. Myeloid‐derived growth factor (MYDGF) is a myeloid cell‐secreted factor with emerging metabolic functions, but its action in diabetes‐associated non‐alcoholic fatty liver disease (DM NAFLD) is undefined. To investigate the influence of myeloid cell‐derived MYDGF on DM NAFLD, both loss‐ and gain‐of‐function approaches were applied in vivo. DM NAFLD models were established in *Mydgf*‐knockout (KO) mice, *Pparg* ^ΔLyz2^ mice and *Pparg* ^ΔLyz2^/KO (DKO) mice. For in vitro studies, primary Kupffer cells (KCs) were stimulated with high glucose and recombinant MYDGF (rMYDGF) and bone marrow cells (BMCs) were co‐cultured with KCs via a transwell system. Protein levels related to DM NAFLD pathology and signalling pathways were quantified. In this study, we found that MYDGF was significantly downregulated in DM NAFLD mouse liver. Myeloid MYDGF deficiency exacerbated hepatic inflammation, lipogenesis, steatosis and macrophage infiltration, while reconstituting myeloid MYDGF reversed these phenotypes. rMYDGF attenuated inflammation, reduced macrophage foaming, decreased cholesterol content and increased efferocytosis. Mechanistically, MYDGF induced AMP‐activated protein kinase (AMPK) phosphorylation, which subsequently activated the peroxisome proliferator‐activated receptor γ (PPARγ)/liver X receptor α (LXRα) pathway in KCs and upregulated downstream ABCA1 and ABCG1 expression. These results identify the AMPK/PPARγ/LXRα‐ABCA1/ABCG1 cascade as the core signalling axis mediating MYDGF's metabolic regulatory effects, which bridges the previously unrecognized functional linkage between myeloid cell function and hepatic lipid homeostasis, and explains the protective phenotype observed in MYDGF‐reconstituted DM NAFLD mice. In conclusion, myeloid cell‐derived MYDGF ameliorates DM NAFLD, decreases cholesterol content in macrophages and reduces lipid accumulation via the AMPK/PPARγ/LXRα pathway. MYDGF serves as a key mediator for bone marrow‐liver metabolic crosstalk, indicating that the bone marrow may act as a potential endocrine target for the prevention and treatment of diabetes‐related metabolic disorders.

## 1 Introduction

Non‐alcoholic fatty liver disease (NAFLD) is the predominant liver disorder worldwide and a major health concern, frequently complicated by hepatic fibrosis and cancer [1, 2]. Obesity and Type 2 diabetes mellitus (DM) are notable drivers of steatosis and NAFLD progression; DM with NAFLD (DM NAFLD) is the most common chronic complication of DM [3]. NAFLD is defined by hepatic steatosis (fat accumulation exceeding 5% of liver composition), wherein lipid uptake and synthesis outweigh fatty acid oxidation, causing lipid accumulation and disease progression [4]. Because DM NAFLD is linked to inflammation, insulin resistance (IR) and lipid abnormalities, it elevates mortality and healthcare costs [5]; however, effective treatments remain unavailable [6].

Resident hepatic macrophages are vital innate immune components modulating immune responses in NAFLD [7, 8]; their recruitment and cytokine release trigger proinflammatory cascades [9]. Hepatic macrophages are classified by origin into resident Kupffer cells (KCs) and bone‐marrow‐derived macrophages (BMDMs) [10]. In DM NAFLD, dysfunctional KCs emit chemokines, driving monocyte infiltration [11]; recruited monocytes replenish the KC pool or become proinflammatory BMDMs [12, 13]. Hyperglycemia in DM induces homing of peripheral blood mononuclear cells to the liver, increasing hepatic macrophages and enhancing low‐density lipoprotein (LDL) uptake, forming “foam cells” [14]; it also stimulates inflammatory mediator and chemokine release, downregulates triggering receptor expressed on myeloid cells 2 (TREM2), belongs to the lipid‐associated macrophage (LAM) population, and impairs efferocytosis, worsening DM NAFLD [15].

Peroxisome proliferator‐activated receptor gamma (PPARγ) governs glucose and lipid homeostasis, inflammation and fibrogenesis [16]. Essential for macrophage development, it regulates maturation and metabolism through transrepression [17] and macrophage polarization via chromatin remodelling independently of ligand binding [18]. Pharmacological PPARγ agonists alleviate liver inflammation and fibrosis via hepatic macrophages in DM NAFLD [19]; however, because these benefits partly stem from promoted adipogenesis and altered lipid distribution [20], macrophage‐specific PPARγ regulation warrants further study. Among ATP‐binding cassette (ABC) transporters, ABCA1 exports free cholesterol to lipid‐poor apolipoprotein A‐I, forming high‐density lipoprotein (HDL) that accepts cholesterol carried by ABCG1 [21, 22, 23]. PPARγ and LXRα are nuclear receptors: upon heterodimerizing with the retinoid X receptor, LXRα binds LXR response elements in the *Abca1* and *Abcg1* promoters, promoting transcription [24]. Activated PPARγ facilitates cholesterol elimination by upregulating LXRs and suppresses NF‐κB signalling; activation of the PPARγ/LXRα cascade promotes macrophage cholesterol efflux via ABCA1 and ABCG1, a potential strategy for atherosclerosis [25, 26]. Nevertheless, PPARγ agonists face difficulties in metabolic disorders owing to adverse effects such as cardiac failure [27].

Bone marrow is a crucial haematopoietic organ whose additional physiological roles, such as endocrine functions, remain unexplored. We previously showed that myeloid‐derived growth factor (MYDGF), released by bone marrow‐derived monocytes and macrophages [28], improved IR and glucose‐lipid metabolism in diabetic mice [29]. Given the strong correlation between metabolic disorders and NAFLD [30, 31], how MYDGF regulates ABCA1 and ABCG1 expression and its role in DM NAFLD remain unclear. AMP‐activated protein kinase (AMPK), upstream of PPARγ, controls lipid and energy metabolism [32, 33]; whether the AMPK/PPARγ/LXRα pathway mediates MYDGF's regulation of ABCA1 and ABCG1 is unknown. In this study, we aimed to assess whether and how MYDGF influences DM NAFLD and mediates liver‐bone marrow crosstalk in liver fat metabolism. We hypothesized that MYDGF could inhibit lipid accumulation in macrophages and reduce the area of hepatic lipid deposition in DM NAFLD through activating the AMPK/PPARγ/LXRα pathway. This study is the first to demonstrate that MYDGF exerts beneficial effects on lipid accumulation in macrophages and DM NAFLD.

## 2 Results

### 2.1 Decreased MYDGF Levels and Increased Inflammation in DM NAFLD Mice

Our results showed that the level of MYDGF in the serum of DM NAFLD mice was obviously reduced compared to the control group (Figure S1A and Table S1). Moreover, the results revealed that the levels of MYDGF protein and *Mydgf* mRNA were reduced in the bone marrow of DM NAFLD mice compared to control mice (Figure S1B–D). These results suggest that there might be a correlation between MYDGF and DM NAFLD.

A crucial element in the worsening of NAFLD is inflammation [8]. Consistently, the concentrations of tumour necrosis factor‐α (TNF‐α), interleukin‐1β (IL‐1β) and IL‐6 in the circulation of DM NAFLD mice were significantly increased compared to those in the control group (Table S1). Similarly, compared with control group mice, the levels of inflammatory factors in the livers of DM NAFLD mice were significantly elevated (Figure S1E). Interestingly, in DM NAFLD mice, serum MYDGF levels showed a negative correlation with multiple serum inflammatory markers (Figure S1F). These findings suggest that there is a link between MYDGF and inflammation in DM NAFLD.

In mice with DM NAFLD, metabolic impairments were identified. These included higher body weight, alanine aminotransferase (ALT), aspartate aminotransferase (AST), total cholesterol (TC), triglycerides (TG), low‐density lipoprotein cholesterol (LDL‐C), serum free fatty acids (FFA), fasting insulin levels and fasting blood glucose (FBG) (Table S1).

### 2.2 Myeloid Cell‐Specific MYDGF Deficiency Exacerbated Inflammation, Lipogenesis, Macrophage Accumulation and Hepatic Steatosis in DM NAFLD Mice

As previously reported, *Mydgf* expression was predominantly eliminated in the entirety of bone marrow cells in *Mydgf* knockout (KO) mice [34]. First, no significant differences were observed after staining the femur sections of KO mice and wild‐type (WT) mice with toluidine blue (Figure S2B), suggesting the preservation of bone marrow integrity following the knockout of *Mydgf* in the marrow of the mice. Second, the expression of MYDGF was predominantly eliminated in the entire marrow cells of knockout mice, particularly in macrophages and monocytes, while lymphocytes and stromal cells in the bone marrow showed minimal variation (Figure S2C). Third, there were no notable variations in MYDGF levels between WT and KO mice in the heart, liver, spleen, lung, or kidney (Figure S2D), verifying the knockout of MYDGF specifically in myeloid cells. Moreover, our aim was to examine how the lack of MYDGF in myeloid cells affects DM NAFLD progression in mice. The hepatic steatosis induced by a high‐fat diet (HFD) is more pronounced in DM KO mice compared to DM WT mice, as indicated by an increased number of lipid droplets in the liver and an expanded area of liver index, liver lipid deposition and hepatic TG content in DM KO mice (Figure 1A–C). Notably, in isolated hepatocytes, the mRNA levels of lipid synthesis genes such as *Srebp1c*, fatty acid synthase (*Fasn*), stearoyl‐CoA desaturase 1 (*Scd1) and* acetyl‐CoA carboxylase 1 (*Acc1*) were markedly higher, while *Pparγ* was lower in DM NAFLD KO mice compared to DM NAFLD WT mice. Nonetheless, the expression levels of mRNA for genes associated with lipid oxidation, like carnitine palmitoyltransferase 1a (*Cpt1a*) and acyl‐CoA oxidase 1 (*Acox1*), demonstrated no significant change between DM NAFLD KO mice and DM NAFLD WT mice (Figure 1D). Moreover, significant liver dysfunction was noted in MYDGF‐deficient DM NAFLD mice, characterized by elevated ALT and AST levels (Figure 1E). These results indicate that the lack of MYDGF in myeloid cells intensifies lipogenesis and liver steatosis in DM NAFLD mice.

**FIGURE 1:**
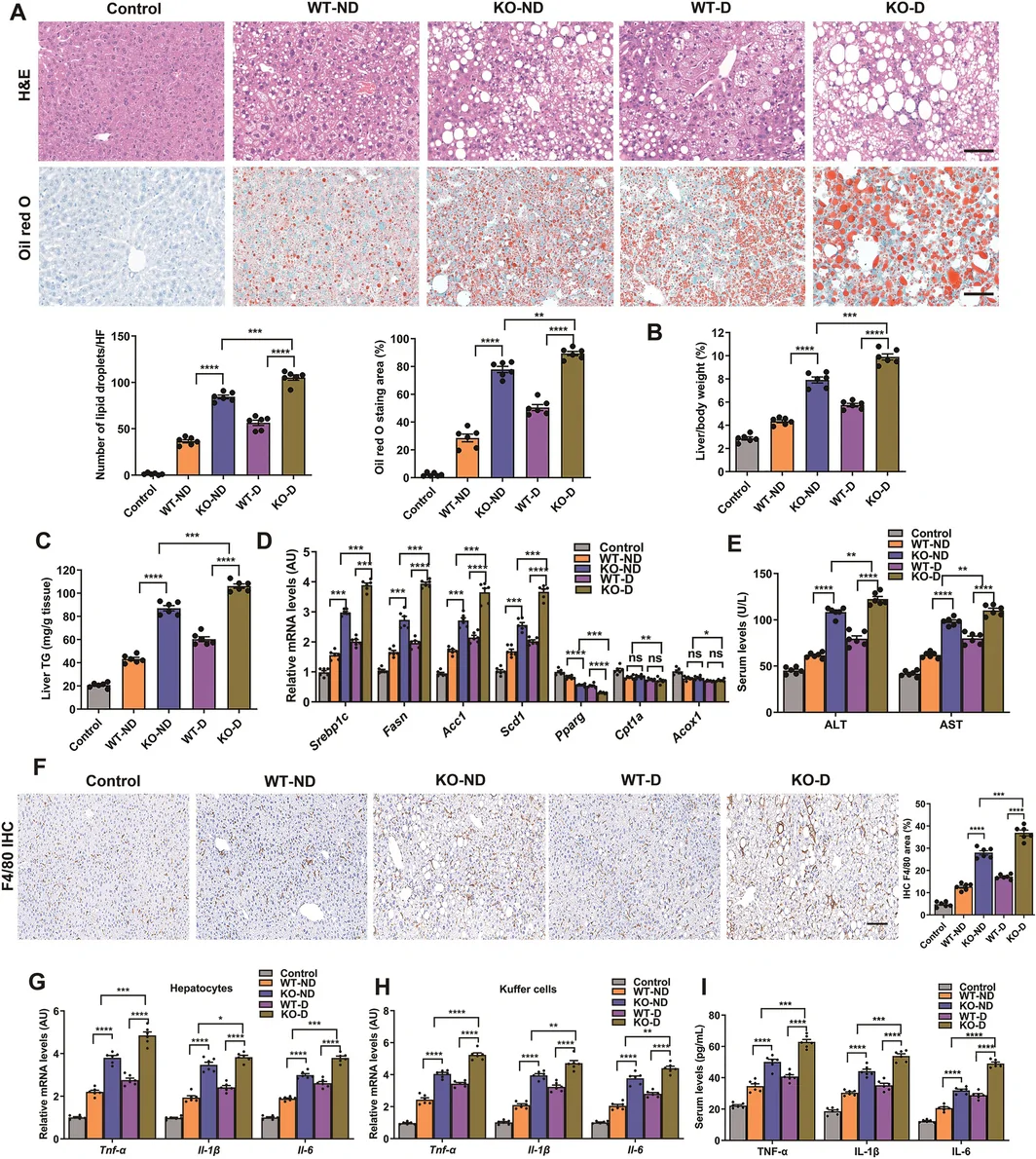
Myeloid cell‐specific MYDGF deficiency exacerbated inflammation, lipogenesis and hepatic steatosis in DM NAFLD mice. HFD/STZ‐induced DM models in WT and KO mice were fed an HFD for 12 weeks. The control group indicates WT mice fed an NCD. (A) Representative images showing HE staining and oil red O staining of liver sections. (B) Liver/body weight. (C) Hepatic TG content. (D) mRNA levels of genes related to fat metabolism in isolated hepatocytes. (E) Serum ALT and AST levels. (F) Representative images showing F4/80 staining of liver sections. (G) Levels of inflammatory factors in isolated hepatocytes. (H) Levels of inflammatory factors in KCs. (I) Levels of inflammatory factors in serum. Data are presented as the mean ± SEM. Black scale bar: 50 μm. *p < 0.05, **p < 0.01, ***p < 0.001, ****p < 0.0001. D, diabetes mellitus mice. ND, non‐diabetic mice. Significant differences were determined by Student's *t*‐test or one‐way ANOVA followed by Tukey's post‐test. *Acc1*, acetyl‐CoA carboxylase 1; *Acox1*, acyl‐coenzyme A oxidase 1; ALT, alanine aminotransferase; AST, aspartate aminotransferase; *Cpt1a*, carnitine palmitoyltransferase 1a; DM, diabetes mellitus; *Fasn*, fatty acid synthase; HFD, high‐fat diet; *Il‐1β*, interleukin‐1β; *Il‐6*, interleukin‐6; KCs, Kupffer cells; KO, *Mydgf* knock out; ND, non‐diabetes mellitus; *Pparg*, peroxisome proliferator‐activated receptor gamma; *Scd1*, stearoyl‐CoA desaturase 1; *Srebp1c*, sterol regulatory element‐binding protein 1c; STZ, streptozotocin; TG, triglyceride; *Tnf‐α*, tumour necrosis factor‐α; WT, wild type.

Subsequently, we investigated the influence of MYDGF deficiency within myeloid cells on inflammatory responses in mice. The result revealed that, compared to WT DM mice, the proportion of macrophages in the liver of MYDGF‐deficient DM mice fed a HFD was significantly increased (Figure 1F). Furthermore, compared with WT DM mice, the levels of inflammatory factors were significantly elevated in hepatocytes, KCs and the serum of DM KO mice fed with HFD (Figure 1G–I). These data collectively indicate that the absence of MYDGF specifically in myeloid cells exacerbated inflammation in DM NAFLD mice.

Furthermore, our results showed that DM NAFLD KO mice presented reduced glucose and insulin tolerance when compared with DM NAFLD WT mice (Figure S3A–D, Table S2), more severe lipid metabolism disruptions (Table S2) and greater body weight gain (Figure S3E, F). These results indicate that the deficiency of MYDGF in myeloid cells disrupts lipid metabolism.

### 2.3 Bone Marrow Transplantation Alleviated Inflammation, Lipogenesis, Macrophage Accumulation and Hepatic Steatosis in DM NAFLD Mice

Our subsequent aim was to assess the extent of hepatic inflammation, de novo lipogenesis and steatosis following myeloid‐targeted MYDGF reconstitution in DM NAFLD KO mice. To this end, we generated bone marrow chimeric mice to probe how MYDGF restoration modulates inflammatory responses and related diabetic NAFLD protective effects. Circulating blood leukocytes were analysed for CD45.1 and CD45.2 expression via flow cytometry, which supported the successful engraftment after bone marrow transplantation (BMT) (Figure S4A, B). At four weeks post bone marrow cells (BMCs) transplantation from WT donors into KO recipient mice, MYDGF was detectable in peripheral blood and sustained at elevated concentrations (Figure S4C). Consistently, serum MYDGF levels in WT recipients reconstituted with WT BMCs appeared higher than those receiving KO BMC transplantation (Figure S4D). Afterward, we investigated the positive influence of BMT on DM NAFLD mice through a set of transplantation experiments (Figure S4E). Transplanting WT BMCs donors into DM NAFLD KO recipients mitigated hepatic steatosis and lipogenesis, lowered macrophage accumulation and reduced inflammatory factor levels in KCs, hepatocytes and serum (Figure 2A–I), and improved the metabolic profiles (Figure S5, Table S3). On the other hand, transplanting KO BMCs into DM NAFLD WT recipients led to the deterioration of NAFLD (Figure 2A–I) alongside metabolic disorders (Figure S5, Table S3). Our research data indicate that MYDGF derived from BMCs plays a significant and effective role in improving liver inflammation, lipogenesis, macrophage accumulation and hepatic steatosis in DM NAFLD mice.

**FIGURE 2:**
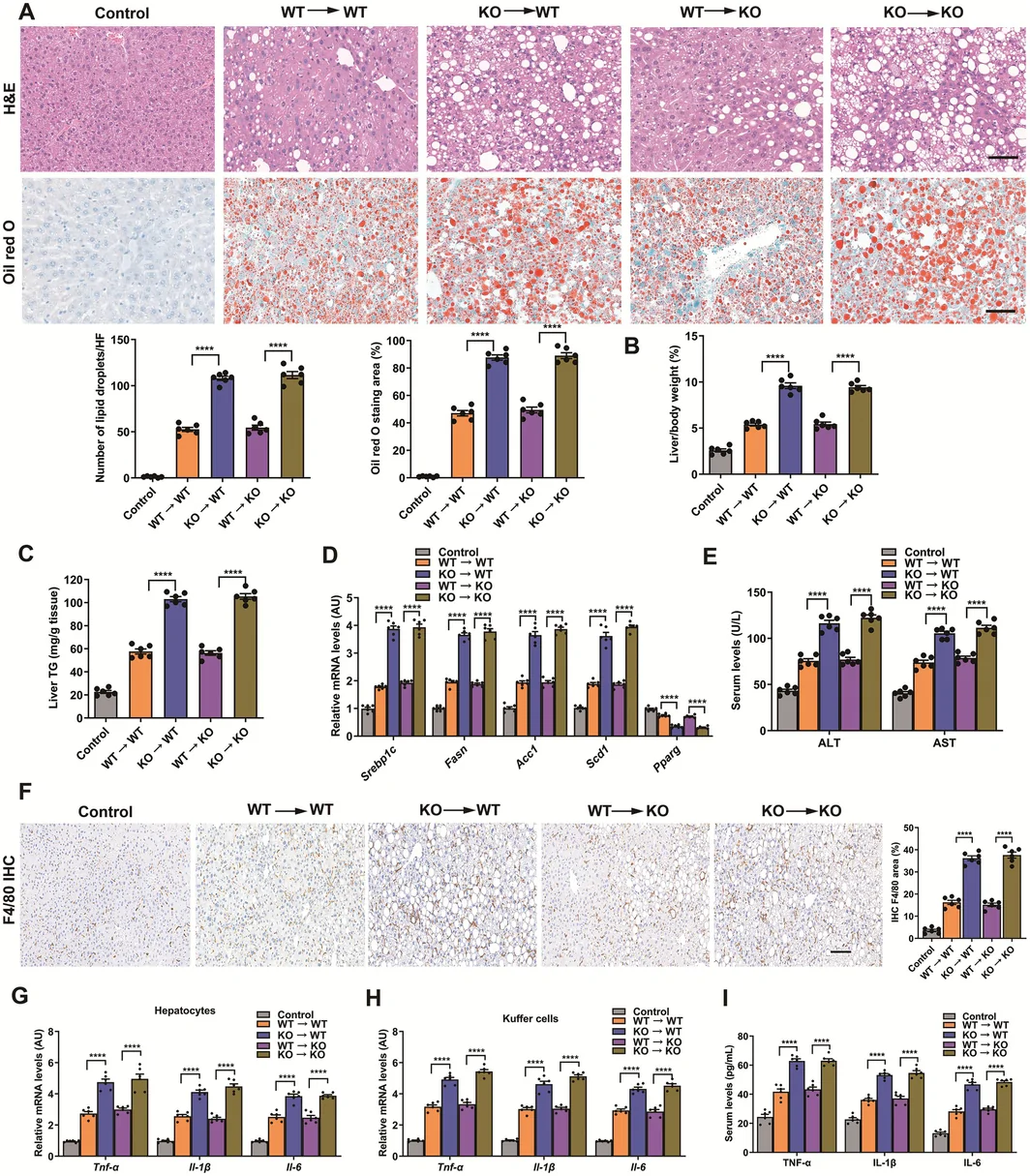
BMT alleviated inflammation, lipogenesis and hepatic steatosis in DM NAFLD mice. BMT was performed in KO and WT mice aged 4 weeks, HFD/STZ‐induced DM models in WT and KO mice, and fed a HFD for 12 weeks as indicated in Figure S4C. WT → WT, WT mice BMCs donors to WT mice recipients. KO → WT, KO mice BMCs donors to WT mice recipients. WT → KO, WT mice BMCs donors to KO mice recipients. KO → KO, KO mice BMCs donors to KO mice recipients. (A) Representative images showing HE staining and oil red O staining of liver sections. (B) Liver/body weight. (C) Hepatic TG content. (D) mRNA levels of genes related to fat metabolism in isolated hepatocytes. (E) Serum ALT and AST levels. (F) Representative images showing F4/80 staining of liver sections. (G) Levels of inflammatory factors in isolated hepatocytes. (H) Levels of inflammatory factors in KCs. (I) Levels of inflammatory factors in serum. Data are presented as the mean ± SEM. Black scale bar: 50 μm. ****p < 0.0001. Significant differences were determined by Student's *t*‐test or one‐way ANOVA followed by Tukey's post‐test. *Acc1*, acetyl‐CoA carboxylase 1; ALT, alanine aminotransferase; AST, aspartate aminotransferase; BMT, bone marrow transplantation; DM, diabetes mellitus; *Fasn*, fatty acid synthase; HFD, high‐fat diet; *Il‐1β*, interleukin‐1β; *Il‐6*, interleukin‐6; KCs, Kupffer cells; KO, *Mydgf* knock out; NAFLD, non‐alcoholic fatty liver disease; ND, non‐diabetes mellitus; *Pparg*, peroxisome proliferator‐activated receptor gamma; *Scd1*, stearoyl‐CoA desaturase 1; *Srebp1c*, sterol regulatory element‐binding protein 1c; STZ, streptozotocin; TG, triglyceride; *Tnf‐α*, tumour necrosis factor‐α; WT, wild type.

### 2.4 Bone Marrow‐Specific Overexpression of MYDGF Improved Inflammation, Lipogenesis, Macrophage Accumulation and Hepatic Steatosis in DM NAFLD Mice

In order to confirm the improvement of hepatic inflammation, lipogenesis and steatosis in DM KO mice through bone marrow‐derived MYDGF restoration, we injected AAV‐MYDGF and AAV‐GFP at a dose of 1 × 10^12^ VP directly into the marrow cavities of DM NAFLD mice. The effectiveness of MYDGF expression after intramedullary administration has been evaluated. Notably, MYDGF levels were able to be detected in the serum just 7 days subsequent to the delivery of AAV‐MYDGF and persisted for as long as 21 days in mice after a single intramarrow treatment (Figure S6A). To keep sustained MYDGF levels in the bone marrow and blood circulation, KO mice were given intramedullary injections of AAV‐MYDGF at three‐week intervals with a total time span of 12 weeks. The findings indicated that serum MYDGF levels remained elevated (Figure S6B). In comparison, at 3 weeks, the levels of MYDGF expression in the bone marrow of mice treated with AAV‐MYDGF were markedly elevated compared to those treated with AAV‐GFP. Conversely, KO mice exhibited minimal MYDGF expression in the liver, heart tissue, lungs and kidneys (Figure S6C, D). The outcomes of the trials depicted in Figure S6E showed that injecting AAV‐MYDGF directly into the bone marrow reduced hepatic steatosis, lowered TG levels, lessened lipogenesis and diminished inflammation (Figure 3A–I), with improved metabolic profiles, particularly in DM KO mice (Figure S7, Table S4). These results indicate that the restoration of MYDGF improved inflammation, lipogenesis, and hepatic steatosis in DM NAFLD mice.

**FIGURE 3:**
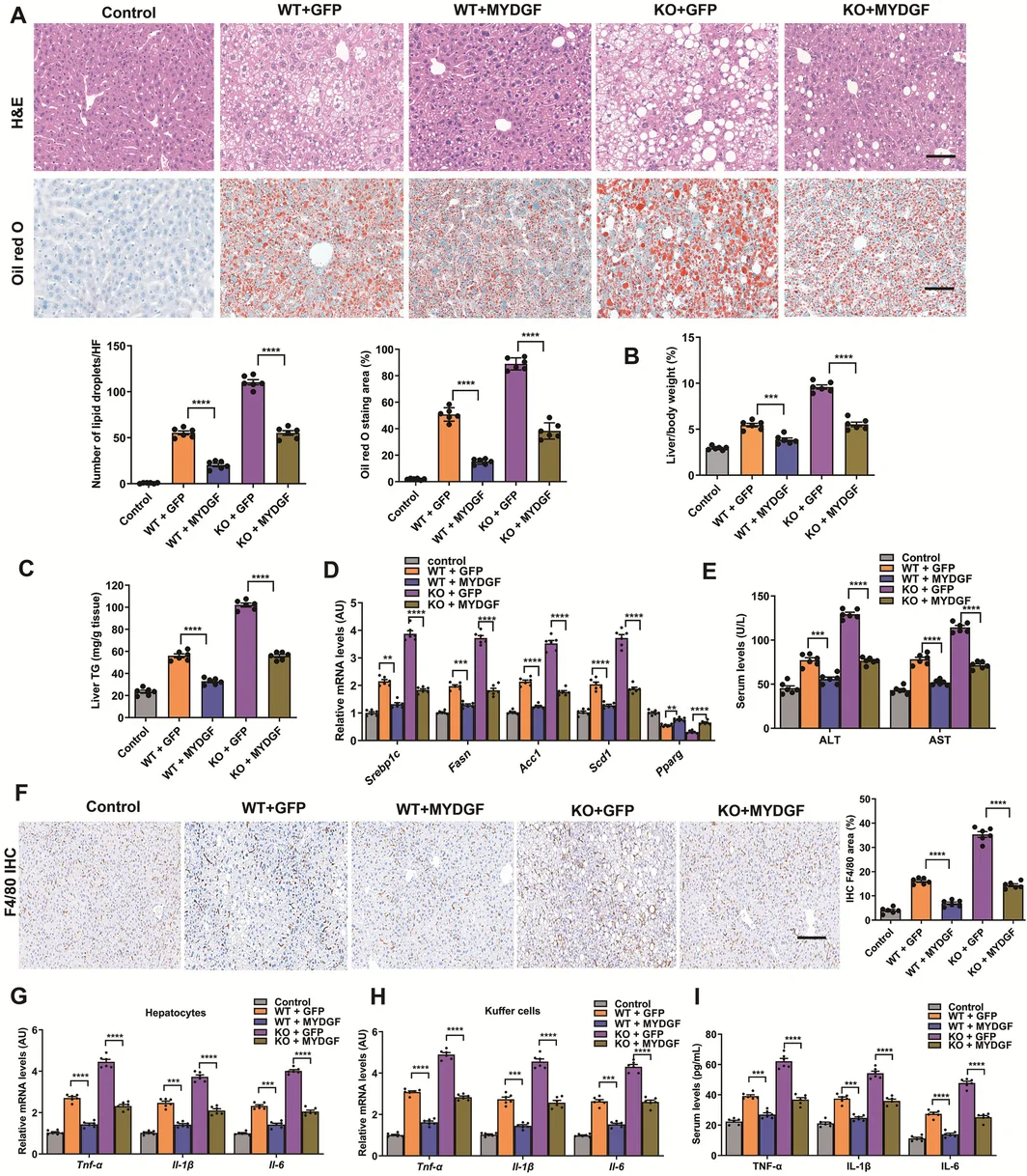
Bone marrow‐specific overexpression of MYDGF alleviated inflammation, lipogenesis and hepatic steatosis in DM NAFLD mice. KO and WT mice aged 4–6 weeks were selected, and intramarrow AAV‐MYDGF injection was done as indicated in Supplementary Figure 6E. (A) Representative images showing HE and oil red O staining of liver sections. (B) Liver/body weight. (C) Hepatic TG content. (D) mRNA levels of genes related to fat metabolism in isolated hepatocytes. (E) Serum ALT and AST levels. (F) Representative images showing F4/80 staining of liver sections. (G) Levels of inflammatory factors in isolated hepatocytes. (H) Levels of inflammatory factors in KCs. (I) Levels of inflammatory factors in serum. Data are presented as the mean ± SEM. Black scale bar: 50 μm.***p < 0.001, ****p < 0.0001. Significant differences were determined by Student's *t*‐test or one‐way ANOVA followed by Tukey's post‐test. *Acc1*, acetyl‐CoA carboxylase 1; ALT, alanine aminotransferase; AST, aspartate aminotransferase; DM, diabetes mellitus; *Fasn*, fatty acid synthase; HFD, high‐fat diet; *Il‐1β*, interleukin‐1β; *Il‐6*, interleukin‐6; KCs, Kupffer cells; KO, *Mydgf* knock out; MYDGF, myeloid‐derived growth factor; NAFLD, non‐alcoholic fatty liver disease; ND, non‐diabetes mellitus; *Pparg*, peroxisome proliferator‐activated receptor gamma; *Scd1*, stearoyl‐CoA desaturase 1; *Srebp1c*, sterol regulatory element‐binding protein 1c; STZ, streptozotocin; TG, triglyceride; *Tnf‐α*, tumour necrosis factor‐α; WT, wild type.

### 2.5 MYDGF Improved Inflammation, Reduced Macrophage Foaming, Decreased Cholesterol Content in Macrophages and Increased Macrophage Efferocytosis

Our current findings reveal that MYDGF prevents macrophage accumulation in DM NAFLD. Here, we examined whether MYDGF directly impacts KCs. We chose treatment with 25 mM glucose and 100 ng/mL recombinant MYDGF (rMYDGF) for 24 h as the most suitable conditions (Figure S8A, B). Afterward, we carried out the experiment shown in Figure S8C. The findings indicated that rMYDGF mitigated the inflammatory response triggered by HG and inhibited the migration of KCs (Figure 4A–C). Besides, rMYDGF reduced macrophage foaming, decreased cholesterol content in macrophages, and increased macrophage efferocytosis function (Figure 4D–F).

**FIGURE 4:**
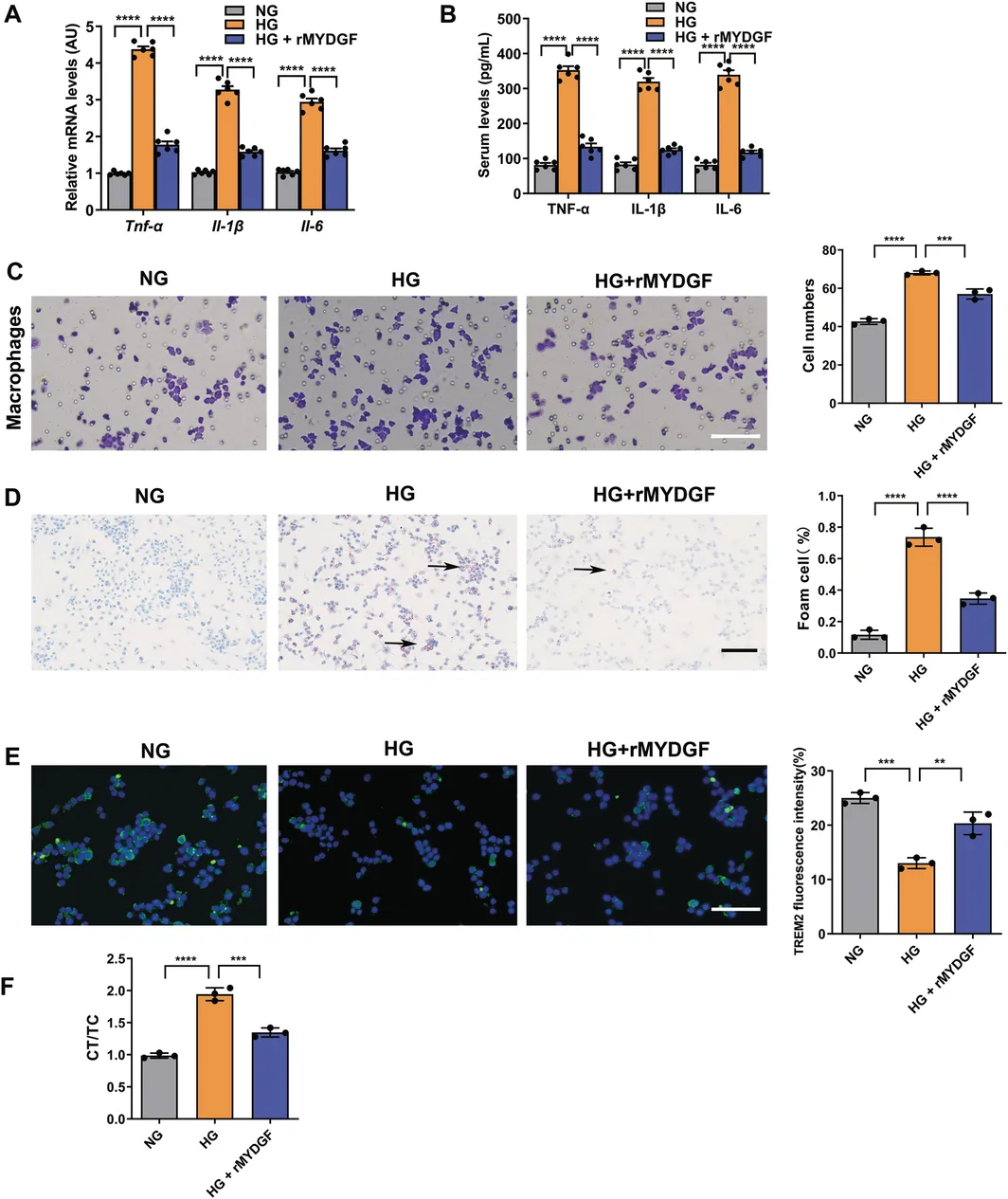
MYDGF improved inflammation, reduced macrophage foaming, decreased cholesterol content in macrophages and increased macrophage efferocytosis in KCs. KCs were treated as described in Figure S8C. (A) The mRNA levels of *Tnf‐α, Il‐1β* and *Il‐6* in KCs after rMYDGF treatments. (B) The levels of inflammatory factors in supernatants after rMYDGF treatments. (C) Representative migration images of macrophages. Scale bar, 100 μm. (D) Representative images showing Oil Red O staining of macrophages. Black arrows indicate foam cells. (E) Representative images showing TREM2 immunofluorescence staining of macrophages. (F) The levels of cholesterol ester/total cholesterol in macrophages. Data are presented as the mean ± SEM. **p < 0.01, ***p < 0.001, ****p < 0.0001. Significant differences were determined by Student's *t*‐test or one‐way ANOVA followed by Tukey's post‐test. CE, cholesterol ester; HG, high glucose; *Il‐1β*, interleukin‐1β; *Il‐6*, interleukin‐6; KCs, Kupffer cells; NG, normal glucose; RMYDGF, recombinant myeloid‐derived growth factor; TC, total cholesterol; *Tnf‐α*, tumour necrosis factor‐α.

Furthermore, we also conducted co‐culture experiments on bone marrow cells (BMCs) and KCs that were exposed to HG (Figure S8D). Co‐culture with WT BMCs notably reduced the mRNA expression of inflammatory factors in KCs, reduced macrophage foaming, decreased cholesterol content in foam cells and increased macrophage efferocytosis function (Figure S9A–F), while co‐culturing with KO BMCs had no impact. Collectively, these findings indicate that MYDGF derived from BMCs protects against DM NAFLD by modulating macrophage function.

### 2.6 The AMPK/PPARγ/LXRα Signalling Pathway Is Involved in the Beneficial Effects of MYDGF in DM NAFLD In Vivo and In Vitro

We continue to explore through which pathways MYDGF might exert protective effects on DM NAFLD. Our research findings reveal that MYDGF inhibits inflammation and macrophage accumulation in the liver. PPARγ is regarded as a crucial transcription factor which has an important function in the regulation and specialization of macrophages. And the PPARγ signalling pathway is one of the most relevant pathways associated with NAFLD [35]. Consistent with previous studies, our experiments also found that the expression of PPARγ in macrophages was obviously increased in the rMYDGF+HG treatment group compared to the HG group (Figure S10A–C). Consequently, the signalling of PPARγ was evaluated both in vitro and in vivo. These results demonstrated that the levels of TREM2, p‐AMPK, PPARγ, LXRα, ABCA1 and ABCG1 in liver macrophages were decreased in DM NAFLD KO mice compared to DM NAFLD WT mice (Figure 5A,B), while the expression of these proteins in liver macrophages was obviously increased in AAV‐MYDGF treated mice compared to AAV‐GFP treated mice (Figure 5C,D). Consistent with the results of in vivo experiments, rMYDGF treatment markedly stimulated HG‐induced alterations in the AMPK/PPARγ/LXRα pathway within liver macrophages (Figure 5E,F). These results show that MYDGF can regulate its downstream ABCA1 and ABCG1 through AMPK/PPARγ/LXRα, participating in cholesterol efflux from liver macrophages to increase efferocytosis. The AMPK/PPARγ/LXRα pathway is crucial for the beneficial effects of MYDGF on DM NAFLD.

**FIGURE 5:**
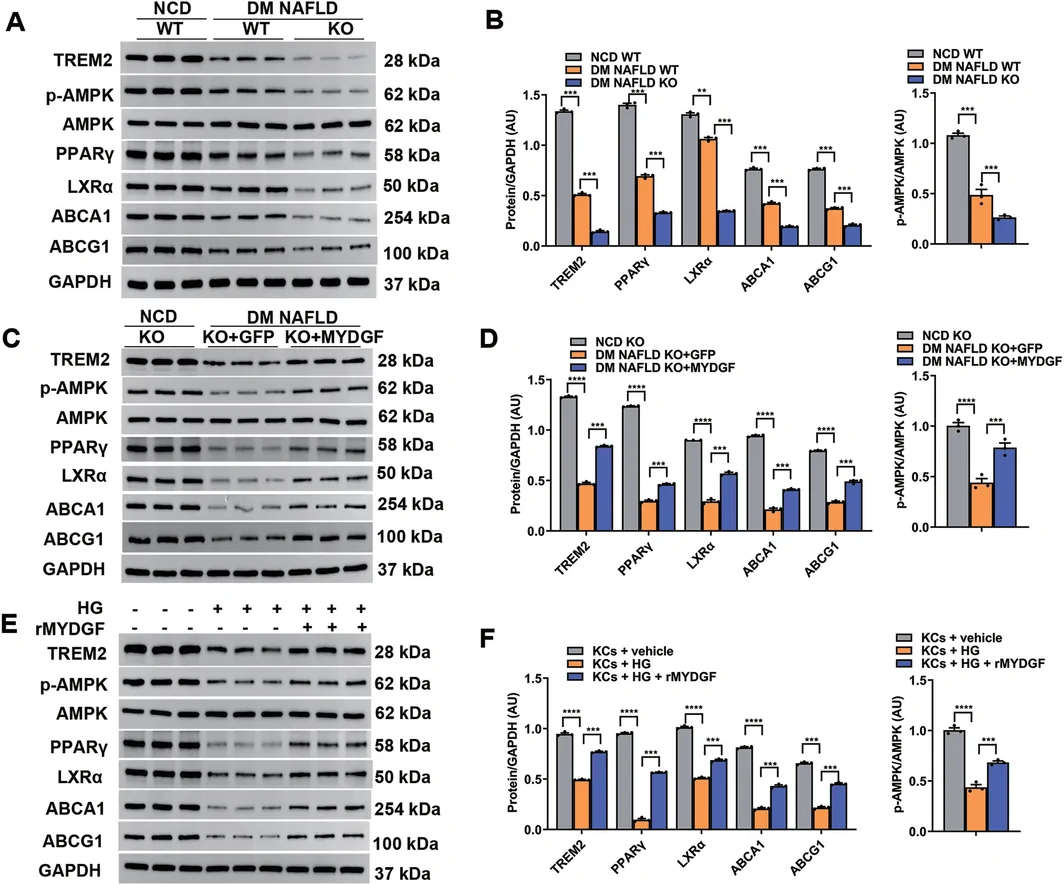
MYDGF promoted the AMPK/PPARγ/LXRα pathway in vivo and in vitro. (A) Levels of AMPK/PPARγ/LXRα pathway proteins in the liver macrophages of KO mice. (B) Quantitative analysis of A. (C) Levels of AMPK/PPARγ/LXRα pathway proteins in AAV‐MYDGF treated liver macrophages of KO mice. (D) Quantitative analysis of C. (E) Levels of AMPK/PPARγ/LXRα pathway proteins in rMYDGF‐treated KCs. (F) Quantitative analysis of E. Data are presented as the mean ± SEM. ***p < 0.001, ****p < 0.0001. Significant differences were determined by Student's *t*‐test or one‐way ANOVA followed by Tukey's post‐test. ABCA1, ATP‐binding cassette transporter A1; ABCG1, ATP‐binding cassette transporter G1; AMPK, AMP‐activated protein kinase; DM, diabetes mellitus; GAPDH, glyceraldehyde‐3‐phosphate dehydrogenase; HG, high glucose; KCs, Kupffer cells; KO, *Mydgf* knockout. NAFLD, non‐alcoholic fatty liver disease; LXRα, liver X receptor α; MYDGF, myeloid‐derived growth factor; NCD, normal chow diet; PPARγ, peroxisome proliferator‐activated receptor gamma; rMYDGF, recombinant myeloid‐derived growth factor; TREM2, triggering receptor expressed on myeloid cells; WT, wild type.

To confirm the effect of AMPK/PPARγ/LXRα signalling in vivo, *Pparg* ^ΔLyz2^ mice were generated by crossing *Pparg* ^fl/fl^ mice with *Lyz2*‐cre mice. First, BMDMs were isolated from *Pparg* ^ΔLyz2^ mice and the effectiveness of *Pparg* gene deletion was verified via RT‐PCR and WB (Figure S11A, B). *Pparg* ^ΔLyz2^ and control mice were mated with *Mydgf* myeloid‐specific KO mice and then male *Pparg* ^ΔLyz2^, *Pparg* ^ΔLyz2^/*Mydgf‐*KO (DKO, double knockout) and control/*Mydgf* KO mice received intramarrow injections of AAV‐MYDGF or AAV‐GFP every three weeks for a 12‐week duration, and DM NAFLD modelling was conducted. First, the *Pparg* ^ΔLyz2^/*Mydgf‐*KO mice were further validated (Figure S11C, D). Then, the formal experiment results showed that, compared with control mice, the expression of TREM2, p‐AMPK, LXRα, ABCA1 and ABCG1 proteins in liver macrophages of *Mydgf‐*KO mice, *Pparg* ^ΔLyz2^ mice and DKO mice was significantly decreased, but to a greater extent in the DKO mice (Figure 6A,B). Compared with the AAV‐GFP‐treated group, the expression of the above proteins in liver macrophages of *Mydgf‐*KO mice, *Pparg* ^ΔLyz2^ mice and DKO mice in the AAV‐MYDGF group was significantly increased, but to a lesser extent in the AAV‐MYDGF treated DKO mice (Figure 6A,B). Compared with control mice, *Mydgf‐*KO mice, *Pparg* ^ΔLyz2^ mice and DKO mice exhibited notably increased hepatic lipid deposition, macrophage accumulation and inflammation, but to a greater extent in the DKO mice (Figure 6C–G). Furthermore, compared with the AAV‐GFP group, the hepatic lipid deposition, macrophage accumulation and inflammation in *Mydgf‐*KO mice, *Pparg* ^ΔLyz2^ mice and DKO mice treated with AAV‐MYDGF were obviously improved, but to a lesser extent in the AAV‐MYDGF treated DKO mice (Figure 6C–G). The findings verified that the AMPK/PPARγ/LXRα pathway is vital for the positive impact of MYDGF on DM NAFLD.

**FIGURE 6:**
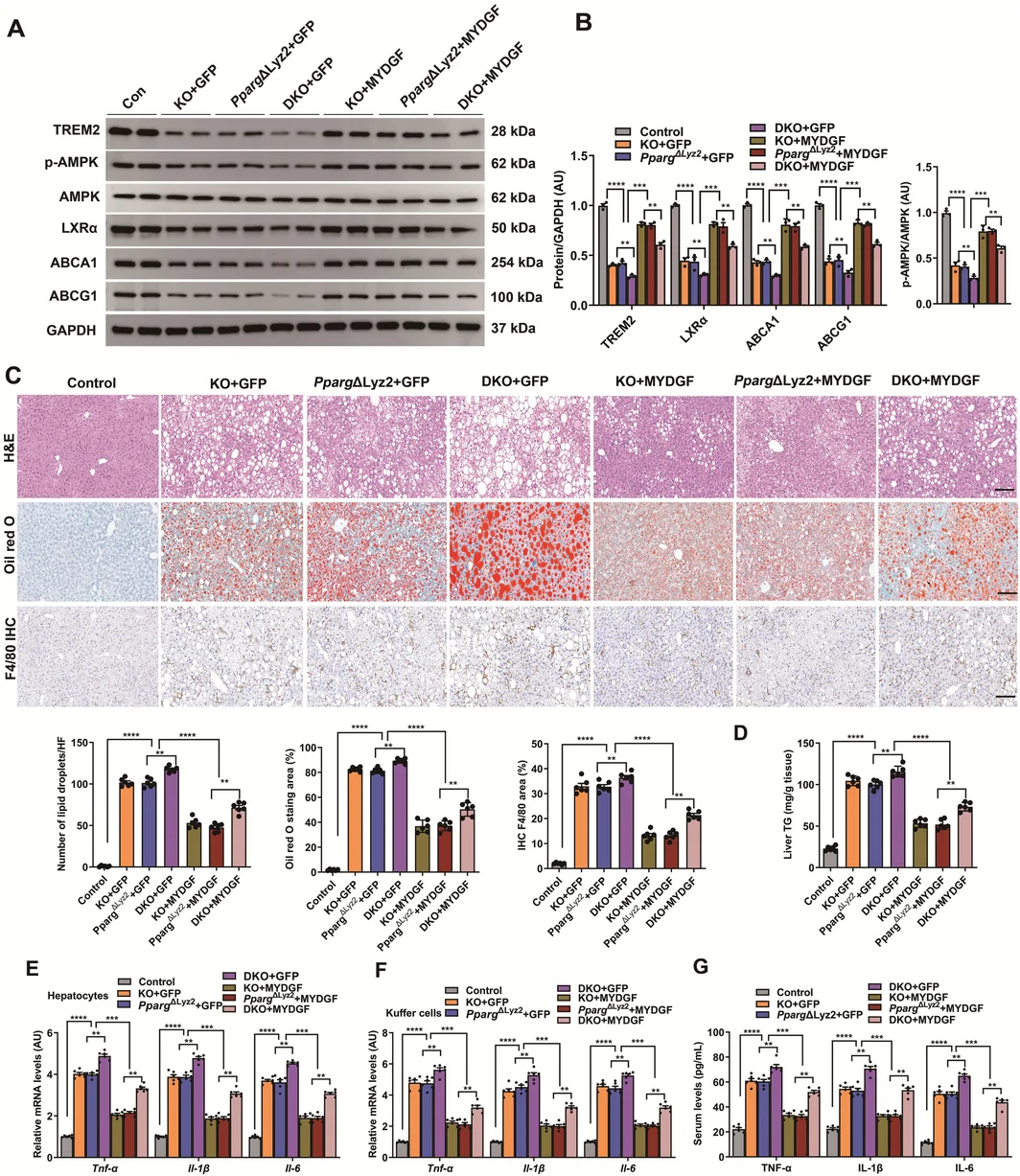
AMPK/PPARγ/LXRα signalling pathway is involved in the beneficial effects of MYDGF in DM NAFLD in vivo. *Pparg* ^ΔLyz2^, *Ppar*g^ΔLyz2^/KO and control/KO mice received intramarrow injections of AAV‐GFP or AAV‐MYDGF every 3 weeks for 12 weeks and DM NAFLD modelling was conducted. (A, B) AMPK/PPARγ/LXRα pathway proteins in the liver macrophages of mice. (C) Representative images showing HE staining, oil red O staining and F4/80 staining of liver sections. Black scale bar: 50 μm. (D) Hepatic TG content. (E) Levels of inflammatory factors in isolated hepatocytes. (F) Levels of inflammatory factors in KCs. (G) Levels of inflammatory factors in serum. Data are presented as the mean ± SEM. **p < 0.01, ***p < 0.001, ****p < 0.0001. Significant differences were determined by Student's *t*‐test or one‐way ANOVA followed by Tukey's post‐test. ABCA1, ATP‐binding cassette transporter A1; ABCG1, ATP‐binding cassette transporter G1; AMPK, AMP‐activated protein kinase; DKO, *Pparg* and *Mydgf* double knock out; DM, diabetes mellitus; GAPDH, glyceraldehyde‐3‐phosphate dehydrogenase; KCs, Kupffer cells; KO, *Mydgf* knock out; LXRα, liver X receptor α; MYDGF, myeloid‐derived growth factor; NAFLD, non‐alcoholic fatty liver disease; PPARγ, peroxisome proliferator‐activated receptor gamma; TREM2, triggering receptor expressed on myeloid cells; WT, wild type.

Next, we aimed to validate the role of the PPARγ pathway in the positive effects of MYDGF using RNA interference‐mediated gene silencing. In the small interfering RNA (siRNA) study, DM NAFLD KO mice were administered a single dose of liver‐specific AAV‐siRNA/*Pparg* (si*Pparg*) or AAV‐siRNA/control (siCon) through the tail vein. As anticipated, the protein and mRNA levels of *Pparg* in the liver were obviously lower in comparison to the siCon‐transfected group after 7 days of intervention (Figure S11E, F). We administered si*Pparg* and AAV‐MYDGF to KO mice and conducted DM NAFLD modelling. As expected, compared with control mice, the expression of TREM2, p‐AMPK, LXRα, ABCA1 and ABCG1 proteins in liver macrophages of KO mice and si*Pparg*‐treated KO mice was significantly decreased, but to a lesser extent in the KO mice (Figure S12A, B). Of note, the increase of TREM2, p‐AMPK, LXRα, ABCA1 and ABCG1 protein levels in liver macrophages by AAV‐MYDGF cannot be fully reversed by knockdown of *Pparg* (Figure S12A, B). Moreover, compared with control mice, KO mice and siPPARγ‐treated KO mice exhibited significantly increased hepatic lipid deposition, macrophage accumulation and inflammation, but to a lesser extent in the KO mice (Figure S12C–G). Subsequently, the decrease in hepatic lipid deposition, macrophage accumulation and inflammation in mice treated with AAV‐MYDGF by MYDGF cannot be fully reversed by knockdown of *Pparg* (Figure S12C–G).

Consistent with the results of in vivo experiments, after 48 h of intervention, si*Pparg* and si*Ampk* effectively silenced the *Pparg* and *Ampk* genes in KC isolated from WT mice (Figure S11G–J). Compared with the vehicle, the expression of TREM2, LXRα, p‐AMPK, ABCG1 and ABCA1 proteins in KCs of HG, si*Pparg*‐treated HG, si*Ampk*‐treated HG and si*Pparg*‐cotreated with si*Ampk* HG groups was significantly decreased, but to a lesser extent in the HG group (Figure 7A,B). Of note, the increase in TREM2, p‐AMPK, LXRα, ABCG1 and ABCA1 protein levels in KCs by MYDGF cannot be fully reversed by knockdown of *Pparg* or *Ampk* (Figure 7A,B). Moreover, compared with the vehicle group, KCs of si*Pparg*‐treated HG, si*Ampk*‐treated HG and si*Pparg*‐cotreated with si*Ampk* HG groups exhibited significantly increased KC adhesion, inflammatory responses, macrophage foaming, macrophage cholesterol content and decreased macrophage efferocytosis function (Figure 7C–F). Subsequently, the decrease in KC adhesion, inflammatory responses, macrophage foaming, macrophage cholesterol content and the increase in macrophage efferocytosis by MYDGF cannot be fully reversed by knockdown of *Ampk* or *Pparg* (Figure 7C–F). These data further confirm that the AMPK/PPARγ/LXRα pathway plays a crucial role in mediating the beneficial effects of MYDGF on HG‐induced KCs (Figure 8).

**FIGURE 7:**
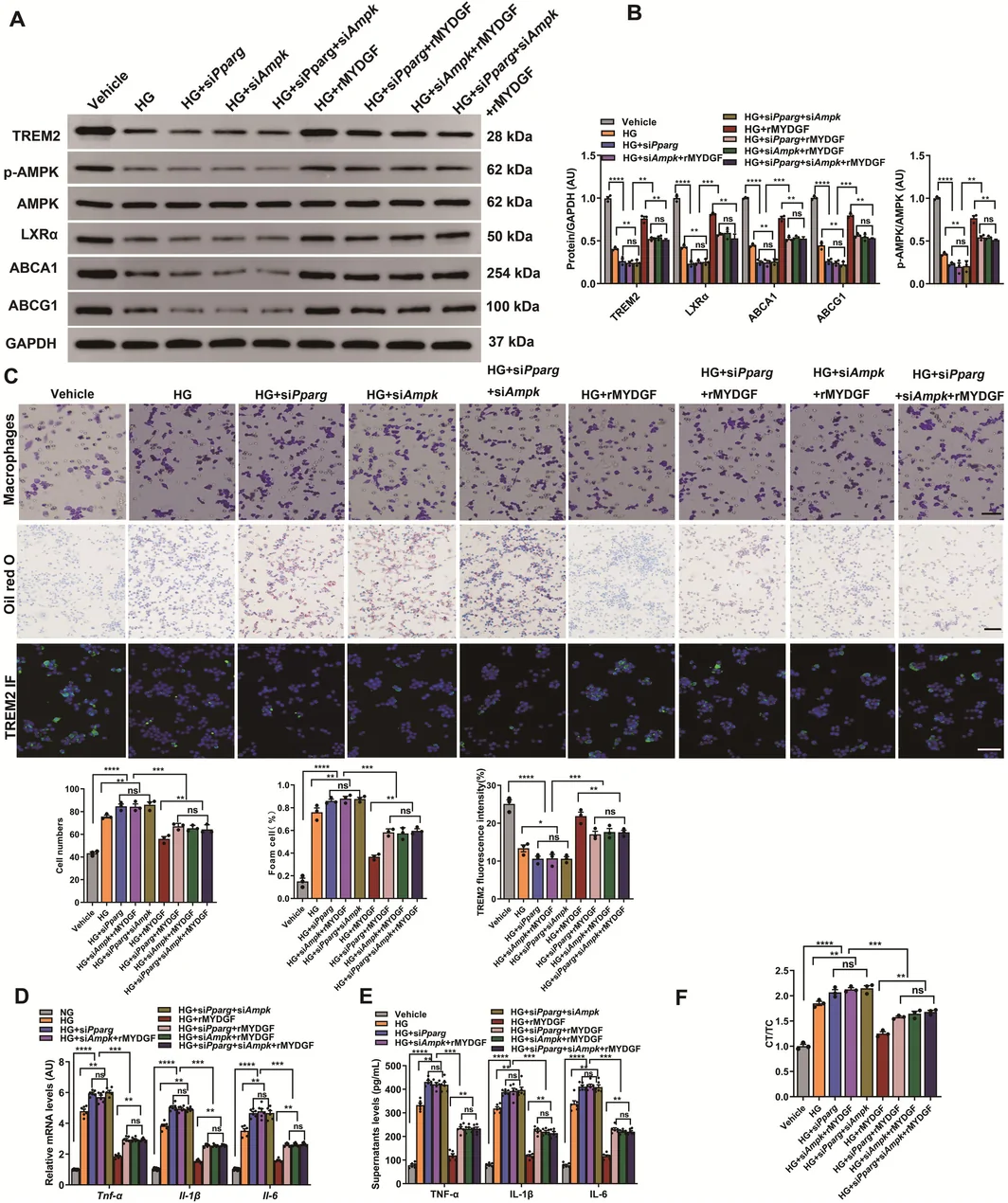
The AMPK/PPARγ/LXRα signalling pathway is involved in the beneficial effects of MYDGF in DM NAFLD in vitro (A, B) AMPK/PPARγ/LXRα pathway proteins in the KCs. (C) Representative migration images of macrophages, Oil red O staining of macrophages and TREM2 immunofluorescence staining of macrophages. Scale bar, 100 μm. Black arrows indicate foam cells. (D) The mRNA levels of *Tnf‐α, Il‐1β* and *Il‐6* in KCs after rMYDGF treatments. (E) The levels of inflammatory factors in supernatants after rMYDGF treatments. (F) The levels of cholesterol ester/total cholesterol in KCs. Data are presented as the mean ± SEM. **p < 0.01, ***p < 0.001, ****p < 0.0001. Significant differences were determined by Student's *t*‐test or one‐way ANOVA followed by Tukey's post‐test. ABCA1, ATP‐binding cassette transporter A1; ABCG1, ATP‐binding cassette transporter G1; AMPK, AMP‐activated protein kinase; GAPDH, glyceraldehyde‐3‐phosphate dehydrogenase; HG, high glucose; KCs, Kupffer cells; LXRα, liver X receptor α; PPARγ, peroxisome proliferator‐activated receptor gamma; rMYDGF, recombinant myeloid‐derived growth factor; TREM2, triggering receptor expressed on myeloid cells.

**FIGURE 8:**
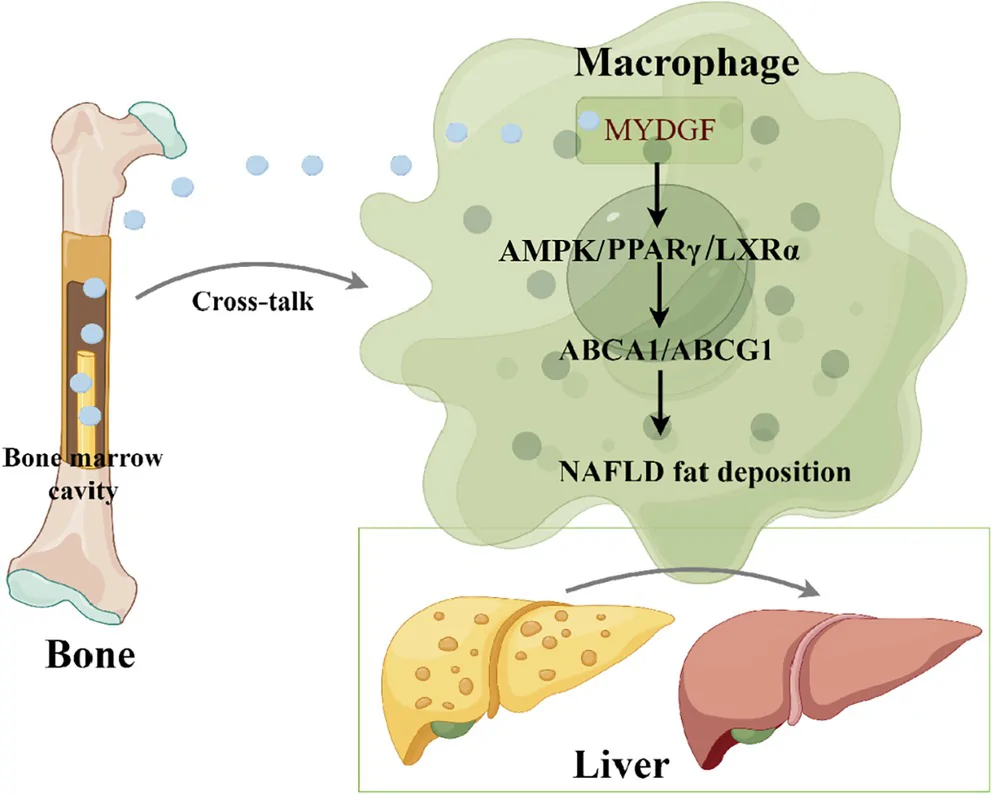
Schematic showing that MYDGF plays a protective role in DM NAFLD via the AMPK/PPARγ/LXRα signalling pathway. ABCA1, ATP‐binding cassette transporter A1; ABCG1, ATP‐binding cassette transporter G1; AMPK, AMP‐activated protein kinase; DM, diabetes mellitus; LXRα, liver X receptor α; MYDGF, myeloid‐derived growth factor; NAFLD, non‐alcoholic fatty liver disease; PPARγ, peroxisome proliferator‐activated receptor gamma.

## 3 Discussion

The main findings of this research can be summed up as follows: (1) MYDGF was down‐regulated in the serum and marrow of DM NAFLD mice; (2) Overexpression of MYDGF levels promoted ABCA1‐ and ABCG1‐mediated cholesterol efflux, suppressed foam cell formation in macrophages, and alleviated the progression of DM NAFLD by activating the AMPK/PPARγ/LXRα signalling pathway. (3) MYDGF may serve as a novel molecular target for the treatment of DM NAFLD. As far as we know, this is the first time that the protective effect of MYDGF in preventing DM NAFLD has been discovered, and the bone marrow has demonstrated an improvement in liver steatosis through MYDGF.

NAFLD presents with several features, such as lipid metabolism disorder, inflammation, liver fibrosis and hepatocyte steatosis [36, 37]. The association between T2DM and NAFLD is complex and mutual, and both often occur jointly as a part of metabolic syndrome. Consequently, the liver has a vital function in modulating abnormal glucose and lipid metabolism [35, 38]. In this study, DM NAFLD mice showed decreased levels of circulating MYDGF along with intensified inflammation, metabolic disorders and impaired liver function. Notably, the deficiency of myeloid‐derived MYDGF intensified the liver index, impaired liver function, and increased lipogenesis and lipid storage in DM NAFLD mice. Conversely, replenishing MYDGF mitigated these effects. Our research indicates that MYDGF has the potential to improve glucose‐lipid metabolism disorder in DM NAFLD. These protective effects further confirm that MYDGF plays a crucial role in guarding DM NAFLD.

Numerous reports have demonstrated that inflammation intensifies hepatic steatosis and initiates the progression of DM NAFLD [39, 40]. Given that macrophages play a key role in shaping the onset and advancement of NAFLD [41], it is essential to investigate how MYDGF in macrophages alters particular characteristics of DM NAFLD. Our research indicated that enhancing MYDGF expression led to elevated levels of high‐density lipoprotein cholesterol (HDL‐C) and reduced levels of LDL‐C, TC and TG. Transfection with AAV‐MYDGF decreased liver dysfunction, hepatic lipid deposition and hepatic macrophage accumulation in DM NAFLD *Mydgf*‐KO mice. Hepatic macrophage foaming was significantly decreased and macrophage efferocytosis function was significantly increased in DM NAFLD *Mydgf*‐KO mice overexpressing MYDGF. These data prove that MYDGF has a positive impact on plasma lipid concentrations and the development of DM NAFLD.

We are also curious about the signalling pathways of MYDGF that contribute to its protective effects in DM NAFLD. ABCA1 and ABCG1 are crucial membrane proteins that assist in the elimination of cholesterol from macrophages, and defective cholesterol efflux by these transporters significantly contributes to the lipid build‐up in macrophages [42]. Our earlier studies have demonstrated that MYDGF is capable of reducing lipid accumulation and preventing NAFLD in animal models [34]. In this study, we observed that transfection with AAV‐MYDGF elevated ABCA1‐ and ABCG1‐mediated cholesterol efflux in DM NAFLD mice. MYDGF also up‐regulated PPARγ, ABCA1 and ABCG1 protein expression, decreased macrophage cholesterol content, and increased macrophage efferocytosis function in KCs. Therefore, promoting ABCG1 and ABCA1‐dependent cholesterol efflux in macrophages is crucial for MYDGF to exert its anti‐lipid accumulation and anti‐DM NAFLD effects.

LXRα is crucial for promoting the transcription of the *Abca1* and *Abcg1* genes. It is widely recognized that PPARγ has the ability to increase the expression of LXRα. Previous studies have reported that PLK1, IFIT2, or danthron activated the PPARγ/LXRα axis, increased ABCA1‐ and ABCG1‐mediated cholesterol efflux, and ultimately inhibited lipid accumulation in macrophages [43, 44, 45]. Similar to these reports, this study confirmed that MYDGF increased the expression of PPARγ and LXRα proteins in liver macrophages of DM NAFLD mice and KCs induced by HG. Furthermore, the improvement of ABCA1 and ABCG1 expression and cholesterol elimination in KCs induced by MYDGF was lessened via siRNA‐mediated down‐regulation of *Pparg*, indicating that the PPARγ/LXRα signalling pathway plays a role in the upregulation of these transporter proteins induced by MYDGF. LXRα is recognized for its ability to boost PPARγ expression, thus establishing a signalling loop between these nuclear receptors, and this interaction results in the augmentation of the signal [43]. When MYDGF is overexpressed, available data suggest that LXRα and PPARγ establish a feedback loop to amplify the pathway's impact, thereby boosting the levels of ABCA1 and ABCG1 protein expression. It is noteworthy that knocking down *Pparg* is unable to completely counteract the rise in ABCA1 and ABCG1 protein levels induced by MYDGF. Therefore, there may be other pathways involved in the expression of ABCA1 and ABCG1 proteins mediated by MYDGF. It has been reported that pharmacological activation of AMPK can upregulate the expression of ABCA1 and ABCG1 proteins, promoting cholesterol efflux in macrophages and thereby reducing atherosclerotic plaques in mice [46]. Administering nobiletin, a bioactive substance found in citrus peels, leads to the phosphorylation of AMPK, thereby activating the PPARγ/LXRα signalling, which increases ABCG1 and ABCA1 protein expression in mice macrophages [42]. Our discoveries also showed that increased MYDGF expression and activation facilitated AMPK phosphorylation in DM NAFLD mice. In the livers of mice that were transfected with AAV‐MYDGF, the level of p‐AMPK also significantly increased. Moreover, suppressing *Ampk* through siRNA significantly reversed the rMYDGF‐induced up‐regulation of LXRα, PPARγ, ABCG1 and ABCA1 protein levels in KCs. Therefore, our discoveries showed that MYDGF boosts the expression of PPARγ, LXRα, ABCG1 and ABCA1, and further promotes cholesterol efflux via AMPK phosphorylation. It is noteworthy that AMPK exhibits several liver‐protective functions, such as anti‐inflammation and anti‐fat deposition [46, 47]. As a multifunctional protective factor, MYDGF may exert hepatoprotective effects against diabetic NAFLD through regulatory mechanisms beyond inhibiting lipid accumulation in macrophages. Beyond the macrophage‐centered hepatoprotective cascade identified in the present study, circulating MYDGF can also directly target hepatocytes and liver sinusoidal endothelial cells to suppress intracellular inflammatory and lipogenic signalling, producing a systemic liver repair effect that operates independently of bone marrow myeloid cell engagement [48, 49]. Our current data suggest that the macrophage‐dependent regulatory axis may be the main pathway through which MYDGF improves the progression of diabetic NAFLD. However, the specific relative contributions of other non‐macrophage‐related cascades still need to be analysed through dedicated follow‐up studies [34, 50].

In summary, we have shown that MYDGF plays a crucial part in inhibiting lipid accumulation in macrophages and the progression of DM NAFLD. These findings rely on the activation of the AMPK/PPARγ/LXRα signalling pathway, which boosts the generation of ABCA1 and ABCG1. Therefore, MYDGF can serve as a drug with great potential for treating DM NAFLD. Furthermore, bone marrow can serve as a potential target for treating metabolic diseases, such as DM, NAFLD and osteoporosis.

## 4 Materials and Methods

### 4.1 Animals and Treatments

*Mydgf*‐floxed (*Mydgf* ^F/F^) mice and *Pparg* ^fl/fl^ mice were obtained from the Shanghai Model Organisms Centre Inc. (Shanghai, China). *Lyz2*‐cre mice were obtained from Jackson Laboratory (Bar Harbour, ME, USA) [34]. *Mydgf*‐floxed mice were crossed with *Lyz2*‐cre mice to generate myeloid cell‐specific *Mydgf* KO and WT mice (Figure S2A) [34]. *Pparg* ^ΔLyz2^ mice were generated by crossing *Pparg* ^fl/fl^ mice with *Lyz2*‐cre mice. *Pparg* ^ΔLyz2^ mice and controls were crossed onto KO mice. Male *Pparg* ^ΔLyz2^/KO and control/KO mice were used for DM NAFLD modelling. All mice were housed in controlled environments, with enclosures at a temperature of (22°C ± 1°C) and a humidity level of 50% [34]. The study protocols received authorization from the Animal Ethics Committee of Hunan Medical College (Approval No. 2024 (A03019)). All animal experimental procedures in this study were divided into six independent but complementary parts, described as follows:

First, to investigate the expression of MYDGF in mice affected by DM NAFLD, male C57BL/6J mice aged 4 to 6 weeks old were randomly split into the DM NAFLD group and the control group. The DM NAFLD group was provided with an HFD (D12492, Research Diets Inc.) for 12 weeks. In contrast, the control group was offered a normal chow diet (NCD) for the same period. After 4 weeks, HFD mice were administered daily intraperitoneal doses of 50 mg/kg streptozotocin (STZ; Sigma‐Aldrich, St. Louis, MO) for five days to generate Type 2 DM mice. Nine days later, the mice were considered DM when their nonfasting blood glucose levels exceeded 13.9 mmol/L for at least two consecutive days. The mice without diabetes were given only citrate buffer and went through the same procedures as the diabetic group.

Second, to investigate the impact of MYDGF deficiency in DM NAFLD mice, male WT and KO mice aged 4 to 6 weeks were subjected to a HFD for 12 weeks. After 4 weeks, HFD mice were treated with the same low‐dose STZ intraperitoneal injection protocol described above to establish the DM NAFLD model.

Third, to investigate the influence of MYDGF originating from BMCs on DM NAFLD mice, 4–6‐week‐old male WT and KO mice received BMCs via tail vein injections from WT or KO donors (WT → WT, KO → WT, WT → KO, KO → KO). After four weeks, all mice were fed a HFD for 12 weeks. After 4 weeks, HFD mice were treated with the same low‐dose STZ intraperitoneal injection protocol described above to establish the DM NAFLD model.

Fourth, to further verify the impact of bone marrow‐derived MYDGF on DM NAFLD mice, male WT and KO mice aged 4 to 6 weeks old were administered four doses of AAV‐MYDGF or AAV‐GFP. Each injection was delivered into the bone marrow at a concentration of 1 × 10^12^ VP. After 4 weeks, all mice were fed a HFD for 12 weeks. After 4 weeks, HFD mice were treated with the same low‐dose STZ intraperitoneal injection protocol described above to establish the DM NAFLD model.

Fifth, male WT and KO mice aged 4 to 6 weeks old were administered a single dose of AAV‐si*Pparg* or AAV‐siCon, with a concentration of 1 × 10^12^ VP in 0.1 mL of phosphate‐buffered saline (PBS, Gibco; Thermo Fisher Scientific Inc.) through the tail vein. All mice were fed a HFD for 12 weeks. After 4 weeks, HFD mice were treated with the same low‐dose STZ intraperitoneal injection protocol described above to establish the DM NAFLD model.

Sixth, male WT and KO mice aged 4 to 6 weeks old were administered a single dose of AAV‐si*Ampk* or AAV‐siCon, with a concentration of 1 × 10^12^ VP in 0.1 mL of PBS through the tail vein. All mice were fed a HFD for 12 weeks. After 4 weeks, HFD mice were treated with the same low‐dose STZ intraperitoneal injection protocol described above to establish the DM NAFLD model.

After the experiment was completed, all the mice were fasted for an entire night. Then, they were sedated by intraperitoneal injection of 60 mg/kg of pentobarbital sodium, and subsequently euthanized humanely to obtain blood and tissue samples.

### 4.2 Construction of AAV Vectors for Mice

The AAV‐MYDGF vector was produced by Hanbio (Shanghai, China). The *Mydgf* gene sequence (GenBank ID: NM_080837.2) was chemically produced within the pHBAAV‐CMV‐MCS‐3flag‐T2A‐ZsGreen construct and then co‐transfected into AAV9‐293 cells together with pAAV‐RC and pHelper plasmids. The AAV encoding liver *Pparg*‐specific siRNA (*Pparg*: GenBank ID NM_000072.7), *Ampk*‐specific siRNA (*Ampk*: GenBank ID NM_000081.7) and the scrambled control siRNA (siCon) were generated by Hanbio. The AAV9 vector system was employed to specifically silence *Pparg* and *Ampk* in the liver. si*Pparg*, si*Ampk* and siCon were incorporated into the AAV9 vector plasmid pSNAV1 and pSNAV1/siRNA was constructed under the control of the constitutive cytomegalovirus promoter. The process for producing, purifying, and preparing large quantities of recombinant AAV has been detailed in earlier work [34].

### 4.3 Glucose and Insulin Tolerance Tests

For the evaluation of intraperitoneal glucose tolerance, the mice were fasted for 12 h prior to receiving glucose (Kelun Pharmaceutical Co., China) at a dosage of 2 g/kg through intraperitoneal injection. For the evaluation of insulin tolerance, mice were subjected to a 6‐h fasting phase and then received a subcutaneous injection of insulin at a dosage of 0.75 U/kg. At the starting point and at 30, 60, 90 and 120 min after the delivery of glucose or insulin, blood was taken from the tail. Glucose levels were measured with a portable glucose monitor (Bayer, Germany). The extra value of the area under the curve was ascertained by using the trapezoidal approach [34].

### 4.4 Mice Biochemical Measurements

The level of MYDGF in the serum was determined using a commercially available reagent kit (Sinobest Biological, Shanghai, China). The fasting serum insulin concentration was measured using electrochemiluminescence immunoassay. The serum levels of IL‐1β, IL‐6 and TNF‐α were determined using an ELISA kit (R&D Systems Inc., Minneapolis, USA). Serum levels of FFA were measured using an enzymatic colorimetric assay kit (Roche Diagnostics, Mannheim, Germany). Serum levels of LDL‐C, HDL‐C, TC, TG, HbA1c, AST and ALT were determined through colorimetric techniques using reagent kits (Jiancheng Bioengineering Institute, Nanjing, China).

### 4.5 Histology

Liver tissues were fixed in 4% formaldehyde, then sectioned and stained with haematoxylin & eosin (HE; Zhongshan Jinqiao, China), or were frozen in liquid nitrogen and stained with oil red O (Amresco, USA), as previously reported [47]. For IHC examination, sections of mouse liver were processed by removing paraffin, rehydrating and then exposed to F4/80 antibody (1:100; CST, #70076). Following initial antibody exposure, the tissue samples were treated with horseradish peroxidase‐linked secondary antibodies targeting immunoglobulins (Jackson Immuno Research, West Grove, USA) and visualization was accomplished using 3,3‐diaminobenzidine. Standard methods were utilized to conduct IF. Macrophages exposed to anti‐mouse TREM2 (1:8000; CST, #55739) and bone marrow samples were processed using MYDGF antibody (1:200; Proteintech, #11353–1‐AP). Upon exposure to primary antibodies, the samples were washed with PBS and treated with suitable fluorescent‐labelled secondary antibodies. Tissue slices were dyed with 4′,6‐diamidino‐2‐phenylindole (DAPI) (Molecular Probes) and inspected by an Olympus FluoView FV1000 confocal microscope (Shinjuku, Japan) [37].

### 4.6 Cell Culture and Treatments

KCs obtained from 8‐week‐old WT mice were cultured in Dulbecco's Modified Eagle's Medium (Gibco; Thermo Fisher Scientific Inc.), supplemented with 10% fetal bovine serum (FBS) (Gibco; Thermo Fisher Scientific Inc.) and 1% antibiotic solution [34]. Regarding the MYDGF intervention experiment, KCs were cultured in DMEM medium under HG induction conditions with different doses of rMYDGF (#10232‐MY‐050, R&D Systems, USA). To explore the potential mechanisms of MYDGF in combating steatosis and inflammation, KCs were pretreated with si*Pparg* or si*Ampk* for 48 h, then 100 ng/mL rMYDGF was added. After 24 h of cultivation, to simulate DM in vivo, KCs were exposed to 25 mM HG, and macrophage foaming, macrophage efferocytosis function and inflammatory profiles were detected.

### 4.7 siRNA Silencing Assay In Vitro

To knockdown *Pparg* or *Ampk* in macrophages, cells were transfected with siRNA against *Pparg* or *Ampk* (GenePharma Co. Ltd., Shanghai, China) using X‐tremeGENE siRNA Transfection Reagent following the manufacturer's instructions. The siRNA oligonucleotides used were as follows: *Pparg* sense sequence: 5′‐GGAUGCAAGGGUUUCUUCCTT‐3′; *Pparγ* antisense sequence: 5′‐ACUCCAC.

AUUACGAAGACATT‐3′; *Ampk* sense sequence: 5′‐UUCUCCGAACGUGUCACG.

UTT′; *Ampk* antisense sequence: 5′‐ACGUGACACGUUCGGAGAATT‐3′. Scrambled siRNA as a negative control.

### 4.8 RT‐PCR Assay

The Applied Biosystems Prism 7000 sequence detection system was used to carry out RT‐PCR, utilizing the specific primers detailed in Table S5. The 2^ΔΔCt^ technique was utilized to assess variations in mRNA expression between different groups. *Gapdh* was used as the internal reference gene for normalizing the expression levels of all target genes. The RT‐PCR thermal cycling started with an initial stage at 95°C for 30 s, followed by 40 cycles of 95°C for 5 s, 60°C for 30 s and 70°C for 30 s.

### 4.9 WB Assay

Antibodies specific to the following proteins were utilized: MYDGF (1:1000; Proteintech, #11353–1‐AP), ABCG1 (1:1000; Abcam, #ab52617), LXRα (1:1000; Abcam, #ab176323), ABCA1 (1:1000; Affinity, #DF8233), TREM2 (1:1000; CST, #55739), phosphorylated‐AMPK (p‐AMPK, 1:1000; CST, #2535), total AMPK (1:1000; CST, #5832), PPARγ (1:1000; Proteintech, #16643–1‐AP) and GAPDH (1:3000; CST, #5174). The utilized secondary antibodies comprised peroxidase‐linked goat anti‐rabbit IgG (1:3000; Jackson ImmunoResearch Laboratories, USA, #111–035‐003) and goat anti‐mouse IgG (1:3000; Jackson ImmunoResearch Laboratories, USA, #115–035‐146). The Western blot signals were captured using a chemiluminescence imaging system (PerkinElmer Inc., Waltham, MA, USA).

### 4.10 BMT Assay

BMCs were isolated from the bone marrow of femurs and tibias of non‐irradiated mice at 4 weeks of age. The recipient mice were lethally irradiated. According to the description of our previous study [34], we transplanted BMCs (1 × 10^6^ cells per mouse) through the tail vein into the recipient mice to generate bone marrow chimerics. In a pilot experiment, CD45.2 recipient mice were lethally irradiated and transplanted with BMCs isolated from congenic CD45.1 donor mice. Eight weeks after transplantation, flow cytometry analysis confirmed that > 85% of peripheral blood leukocytes were CD45.1^+^, indicating successful full donor haematopoietic reconstitution. The antibodies used for staining were monoclonal anti‐CD45.1 (clone A20, #561871, BD Biosciences) and monoclonal anti‐CD45.2 (clone 104, #560695, BD Biosciences).

### 4.11 Injection of AAV Into the Bone Marrow Cavity

Mice were given 5 μL doses of AAV‐MYDGF or AAV‐GFP (1 × 10^12^ VP/mL) through periosteal injection into the femoral medullary cavity every three weeks throughout a period of 12 weeks [34].

### 4.12 Measurement of Oil Red O Staining

KCs were treated with HG for 24 h. At 1 h prior to collection, the cells were stained with Oil Red O (Amresco, USA) to evaluate the quantity of foam cells, as described in previous studies [34, 47].

### 4.13 Cholesterol Content Analysis

KCs were treated with HG for 24 h, and the cells were collected. The cholesterol content was determined using the commercial tissue cell‐free cholesterol enzyme assay kit and tissue cell total cholesterol enzyme assay kit produced by Applygen Technologies Inc. The absorbance readings were recorded using a microplate reader. Finally, the cholesterol ester content was determined by subtracting the free cholesterol content from the total cholesterol content, and the cholesterol ester/to total cholesterol ratio was calculated.

### 4.14 Measurements of Cytokine Levels in Supernatants

The supernatant levels of TNF‐α, IL‐6 and IL‐1β in Kupffer cells were measured by ELISA, following the instructions of the manufacturer (R&D Systems Inc., Minneapolis, USA).

### 4.15 Measurements of Liver TG Levels

Frozen liver samples were analysed for TG concentrations with an assay kit provided by Wako (Richmond, VA). The results are presented in milligrams of TG per gram of liver tissue on a wet weight basis.

### 4.16 Blood Pressure and Other Parameters

SBP and diastolic blood pressure were recorded without invasive procedures utilizing the tail‐cuff technique (Softron BP‐98A, Tokyo, Japan). Blood pressure readings were obtained by averaging results from three consecutive measurements while in a stable state. Food intake, lipid content in faeces, and faecal output were detected according to established procedures [34, 47].

### 4.17 Statistical Analysis

All quantitative data are presented as the mean ± standard error of the mean (SEM). Statistical analyses were performed using IBM SPSS Statistics 27.0 (IBM Corp., Armonk, NY, USA). Prior to comparison, the normality of all datasets was verified using the Shapiro–Wilk test. Pearson correlations were used to identify correlations between variables. For two‐group comparisons, the unpaired Student's *t*‐test was used; for comparisons of three or more independent groups, one‐way analysis of variance (ANOVA) followed by Tukey's post hoc multiple comparisons test was conducted. *p* Value < 0.05 was considered significant.

## Author Contributions

**Zeyu Chen:** methodology, writing – original draft. **Biying Meng:** methodology, data curation. **Bingyi Wang:** methodology. **Yan Ding:** data curation, conceptualization, funding acquisition, writing – review and editing.

## Funding

This work was supported by grants from the National Natural Science Foundation of China (82501017, 82300931) and the Doctoral Scientific Research Foundation of Hunan Medical College (2020122001).

## Conflicts of Interest

The authors declare no conflicts of interest.

## Supporting information


**Figure S1:** Reduced MYDGF levels and enhanced inflammation in NAFLD mice.
**Figure S2:** Bone marrow integrity and the expression of MYDGF in WT and KO mice.
**Figure S3:** Myeloid cell‐specific MYDGF deficiency deteriorated the metabolic profiles of DM NAFLD mice.
**Figure S4:** The expression of MYDGF after BMT in mice.
**Figure S5:** BMT improved metabolic profiles in DM NAFLD mice.
**Figure S6:** The expression of MYDGF after intramarrow injection of AAV‐MYDGF in mice.
**Figure S7:** Bone marrow‐specific overexpression of MYDGF improved metabolic profiles in DM NAFLD mice.
**Figure S8:** Identification of the optimum conditions for the incubation of KCs with HG and rMYDGF.
**Figure S9:** MYDGF improved inflammation, reduced macrophage foaming, reduced cholesterol content in macrophages and enhanced macrophage efferocytosis in co‐culture experiments.
**Figure S10:** Expression of PPARγ protein and mRNA in two groups of macrophages.
**Figure S11:** The confirmation of Pparg knockdown in PpargΔLyz2 mice and PpargΔLyz2/KO mice or silencing in liver in vitro, as well as the confirmation of Pparg or Ampk knockdown after siCon and siPparg or siAmpk treatment in KCs.
**Figure S12:** AMPK/PPARγ/LXRα signalling pathway is involved in the beneficial effects of MYDGF in DM NAFLD in vivo.
**Table S1:** Biochemical characteristics in control and DM NAFLD mice at the end of the experiments.
**Table S2:** Effects of bone marrow‐specific MYDGF deficiency on biochemical characteristics in WT and KO DM NAFLD mice at the end of the study.
**Table S3:** Effects of MYDGF restoration by BMT on biochemical characteristics in WT and KO DM mice at the end of the study.
**Table S4:** Effects of MYDGF restoration by intramarrow injection on biochemical characteristics in WT and KO DM mice at the end of the study.
**Table S5:** A list of RT‐PCR primers used in this study.

## Data Availability

The data that support the findings of this study are available from the corresponding author upon reasonable request.
