# Supplementary material for: Myeloid‐Derived Growth Factor Alleviates Diabetic Non‐Alcoholic Fatty Liver Disease by Up‐Regulating ABCA1 and ABCG1 Expression via the AMPK/PPARγ/LXRα Pathway
Source: J Cell Mol Med. 2026 Nov 1;30(21):e71428. doi: 10.1111/jcmm.71428 (PMC13725103; doi:10.1111/jcmm.71428)
Supplement: Supplementary file 1 [file JCMM-30-e71428-s001.pdf]

Table S1 Biochemical characteristics in control and DM NAFLD mice at the end of the experiments.

Variables	Control	DM NAFLD	*P* values
N	10	10	-
Body weight (g)	25.82 ± 0.41	31.50 ± 0.68	< 0.001
SBP (mmHg)	103.50 ± 1.33	105.40 ± 1.47	0.350
DBP (mmHg)	73.60 ± 0.82	75.10 ± 1.14	0.299
FBG (mmol/L)	5.08 ± 0.10	14.40 ± 0.17	< 0.001
HbA1c (%)	5.23 ± 0.20	13.68 ± 0.41	< 0.001
Insulin (mU/L)	9.88 ± 0.43	20.78 ± 0.36	< 0.001
TC (mmol/L)	3.14 ± 0.08	5.43 ± 0.18	< 0.001
TG (mmol/L)	1.09 ± 0.07	2.89 ± 0.09	< 0.001
LDL-C (mmol/L)	1.21 ± 0.09	2.38 ± 0.08	< 0.001
HDL-C (mmol/L)	1.51 ± 0.11	1.01 ± 0.06	< 0.001
FFA (mmol/L)	0.54 ± 0.03	0.98 ± 0.04	< 0.001
ALT (U/L)	45.00 ± 2.64	61.50 ± 2.39	< 0.001
AST (U/L)	41.53 ± 1.46	59.20 ± 1.63	< 0.001
TNF-α (pg/mL)	21.16 ± 0.90	37.18 ± 0.94	< 0.001
IL-1β (pg/mL)	26.52 ± 0.81	33.85 ± 0.89	< 0.001
IL-6 (pg/mL)	14.20 ± 0.39	23.63 ± 0.98	< 0.001
MYDGF (pg/mL)	184.70 ± 3.28	115.30 ± 4.09	< 0.001

Data are expressed as mean ± SEM. Significant differences were determined by

Student's *t*-test.

Abbreviations: SBP, systolic blood pressure. DBP, diastolic blood pressure. FBG, fasting blood glucose. HbA1C, hemoglobin A1c. TC, cholesterol. TG, triglycerides. LDL-C, low-density lipoprotein-cholesterol. HDL-C, high-density lipoprotein-cholesterol. FFA, free fatty acid. ALT, alanine aminotransferase. AST, aspartate aminotransferase. TNF-α, tumor necrosis factor-α. IL-1β, interleukin-1β. IL-6, interleukin-6. MYDGF, myeloid-derived growth factor. DM, diabetes mellitus.

Table S2 Effects of bone marrow-specific MYDGF deficiency on biochemical characteristics in WT and KO DM NAFLD mice at the end of the study.

Variables	Control	WT-ND	KO-ND	WT-D	KO-D
N	6	6	6	6	6
Body weight (g)	27.14 ± 0.38	31.39 ± 0.69	35.32 ± 0.26$^{\#}$	34.95 ± 0.40$^{\#}$	37.82 ± 0.39$^{\#*}$
Food intake (g/week)	20.07 ± 0.95	20.30 ± 0.68	20.99 ± 0.53	21.18 ± 0.62	21.69 ± 0.52
Fecal output (g/week)	2.30 ± 0.07	2.23 ± 0.08	2.22 ± 0.07	2.28 ± 0.11	2.25 ± 0.10
Lipid content in feces (mg/g)	24.42 ± 0.55	41.85 ± 0.77	42.00 ± 0.84	45.12 ± 0.70$^{\#}$	46.06 ± 0.80$^{\#}$
SBP (mmHg)	108.00 ± 2.50	107.83 ± 1.78	103.16 ± 1.68	104.17 ± 1.92	105.50 ± 1.71
DBP (mmHg)	72.50 ± 1.26	73.50 ± 1.57	72.33 ± 1.22	74.83 ± 1.14	73.50 ± 1.54
FBG (mmol/L)	5.20 ± 0.12	5.24 ± 0.08	5.34 ± 0.12	14.46 ± 0.37$^{\#}$	17.24 ± 0.47$^{\#*}$
HbA1c (%)	5.09 ± 0.07	5.08 ± 0.07	5.23 ± 0.07	13.77 ± 0.37$^{\#}$	16.78 ± 0.44$^{\#*}$
Insulin (mU/L)	8.41 ± 0.33	14.18 ± 0.78	19.91 ± 0.33$^{\#}$	20.62 ± 0.52$^{\#}$	23.08 ± 0.75$^{\#*}$
TC (mmol/L)	3.18 ± 0.08	4.39 ± 0.08	6.51 ± 0.20$^{\#}$	6.25 ± 0.08$^{\#}$	8.62 ± 0.35$^{\#*}$
TG (mmol/L)	1.00 ± 0.05	2.66 ± 0.08	3.61 ± 0.08$^{\#}$	3.44 ± 0.06$^{\#}$	5.41 ± 0.12$^{\#*}$
FFA (mmol/L)	0.40 ± 0.02	0.82 ± 0.04	1.57 ± 0.05$^{\#}$	1.58 ± 0.04$^{\#}$	2.10 ± 0.06$^{\#*}$
LDL-C (mmol/L)	1.02 ± 0.04	1.78 ± 0.05	2.27 ± 0.08$^{\#}$	2.18 ± 0.07$^{\#}$	3.15 ± 0.06$^{\#*}$
HDL-C (mmol/L)	1.42 ± 0.03	1.32 ± 0.04	1.07 ± 0.06$^{\#}$	1.12 ± 0.06$^{\#}$	0.84 ± 0.05$^{\#*}$
MYDGF (pg/mL)	179.36± 3.71	117.17 ± 2.86	7.73 ± 0.51$^{\#}$	94.54 ± 4.01$^{\#}$	5.81 ± 0.52$^{\#*}$

Data are expressed as mean ± SEM. HFD/STZ-induced DM models in WT and KO mice, fed a HFD for 12 weeks. The control group indicates WT mice fed a NCD. $^{\#}P <$

0.01 vs. WT ND. $^{*}P$ < 0.01 vs. WT D. One-way ANOVA followed by Tukey's post-hoc test for multiple comparisons was used for groups of three or more.

Abbreviations: SBP, systolic blood pressure. DBP, diastolic blood pressure. FBG, fasting blood glucose. HbA1C, hemoglobin A1c. TC, cholesterol. TG, triglycerides. FFA, free fatty acid. LDL-C, low-density lipoprotein cholesterol. HDL-C, high-density lipoprotein cholesterol. MYDGF, myeloid-derived growth factor. D, diabetes mellitus mice. ND, non-diabetic mice.

Table S3 Effects of MYDGF restoration by BMT on biochemical characteristics in WT and KO DM mice at the end of the study.

Variables	Control	WT→WT	KO→WT	WT→KO	KO→KO
N	6	6	6	6	6
Body weight (g)	27.44 ± 0.44	32.86 ± 0.32	36.79 ± 0.35$^{\#}$	33.60 ± 0.41	37.63 ± 0.38*
Food intake (g/week)	20.53 ± 0.50	21.04 ± 0.68	21.82 ± 0.58	20.56 ± 0.50	20.98 ± 0.89
Fecal output (g/week)	2.14 ± 0.06	2.22 ± 0.05	2.21 ± 0.07	2.17 ± 0.04	2.15 ± 0.05
Lipid content in feces (mg/g)	23.86 ± 0.90	44.02 ± 0.94	44.20 ± 0.52	43.61 ± 1.07	43.42 ± 0.82
SBP (mmHg)	102.00± 1.57	101.83 ± 1.30	102.50 ± 1.20	101.83 ± 1.40	103.50 ± 1.38
DBP (mmHg)	73.17 ± 1.25	75.82 ± 1.35	74.33 ± 1.33	74.67 ± 1.61	74.67 ± 1.31
FBG (mmol/L)	5.05 ± 0.08	11.65 ± 0.33	17.21 ± 0.38$^{\#}$	12.73 ± 0.48	18.07 ± 0.51*
HbA1c (%)	5.14 ± 0.07	10.47 ± 0.21	17.01 ± 0.48$^{\#}$	11.59 ± 0.33	17.29 ± 0.42*
Insulin (mU/L)	8.35 ± 0.12	15.89 ± 0.41	23.21 ± 0.62$^{\#}$	16.95 ± 0.43	23.94 ± 0.57*
TC (mmol/L)	3.24 ± 0.06	5.30 ± 0.39	8.31 ± 0.24$^{\#}$	5.43 ± 0.41	8.78 ± 0.25*
TG (mmol/L)	0.98 ± 0.03	3.04 ± 0.09	5.57 ± 0.13$^{\#}$	3.21 ± 0.09	5.67 ± 0.19*
FFA (mmol/L)	0.38 ± 0.01	1.11 ± 0.06	2.15 ± 0.08$^{\#}$	1.16 ± 0.04	2.48 ± 0.12*
LDL-C (mmol/L)	1.01 ± 0.04	1.63 ± 0.08	3.07 ± 0.06$^{\#}$	1.72 ± 0.05	3.08 ± 0.04*
HDL-C (mmol/L)	1.42 ± 0.05	1.16 ± 0.04	0.83 ± 0.03$^{\#}$	1.09 ± 0.03	0.80 ± 0.04*
MYDGF (pg/mL)	181.96± 3.84	106.01± 2.07	20.52 ± 0.71$^{\#}$	103.41 ± 2.30	5.81 ± 0.48*

Data are expressed as mean ± SEM. $^{\#}P < 0.01$ vs. WT→WT. $^{*}P < 0.01$ vs. WT→KO. One-way ANOVA followed by Tukey's post-test for multiple comparisons was used for groups of three or more.

Abbreviations: SBP, systolic blood pressure. DBP, diastolic blood pressure. FBG, fasting blood glucose. HbA1C, hemoglobin A1c. TC, cholesterol. TG, triglycerides. FFA, free fatty acid. LDL-C, low-density lipoprotein cholesterol. HDL-C, high-density lipoprotein cholesterol. MYDGF, myeloid-derived growth factor.

Table S4 Effects of MYDGF restoration by intramarrow injection on biochemical characteristics in WT and KO DM mice at the end of the study.

Variables	Control	WT + GFP	WT + MYDGF	KO + GFP	KO + MYDGF
N	6	6	6	6	6
Body weight (g)	27.89 ± 0.34	33.33 ± 0.31	30.31 ± 0.35$^{\#}$	37.44 ± 0.48	32.81 ± 0.31*
Food intake (g/week)	20.50 ± 0.70	21.89 ± 0.34	21.36 ± 0.47	21.13 ± 0.48	21.50 ± 0.52
Fecal output (g/week)	2.02 ± 0.07	2.10 ± 0.03	2.12 ± 0.07	2.10 ± 0.05	2.14 ± 0.04
Lipid content in feces (mg/g)	22.49 ± 0.42	44.65 ± 0.62	44.79 ± 0.52	45.33 ± 0.67	45.25 ± 1.02
SBP (mmHg)	105.50 ± 2.33	110.67 ± 2.93	105.17 ± 1.99	113.83 ± 1.66	108.67 ± 1.92
DBP (mmHg)	74.33 ± 1.41	77.83 ± 2.30	73.83 ± 1.45	80.00 ± 2.60	75.50 ± 1.61
FBG (mmol/L)	5.07 ± 0.23	14.09 ± 0.52	11.23 ± 0.51$^{\#}$	16.93 ± 0.25	12.33 ± 0.26*
HbA1c (%)	5.26 ± 0.18	12.99 ± 0.57	7.94 ± 0.31$^{\#}$	16.70 ± 0.45	10.70 ± 0.37*
Insulin (mU/L)	8.08 ± 0.32	20.73 ± 0.42	13.50 ± 1.20$^{\#}$	23.97 ± 1.00	18.08 ± 0.68*
TC (mmol/L)	3.19 ± 0.06	6.02 ± 0.15	3.93 ± 0.16$^{\#}$	8.41 ± 0.20	5.64 ± 0.18*
TG (mmol/L)	1.05 ± 0.06	3.37 ± 0.11	1.23 ± 0.07$^{\#}$	5.42 ± 0.13	3.10 ± 0.11*
FFA (mmol/L)	0.48 ± 0.03	1.49 ± 0.10	0.61 ± 0.05$^{\#}$	2.11 ± 0.06	1.38 ± 0.08*
LDL-C (mmol/L)	0.99 ± 0.02	2.10 ± 0.07	1.27 ± 0.04$^{\#}$	3.13 ± 0.05	2.00 ± 0.04*
HDL-C (mmol/L)	1.46 ± 0.03	1.13 ± 0.03	1.40 ± 0.06$^{\#}$	0.90 ± 0.03	1.24 ± 0.04*
MYDGF (pg/mL)	181.82± 4.36	112.93± 3.24	168.79± 4.16$^{\#}$	6.23 ± 0.49	157.26±3.22*

Data are expressed as mean ± SEM. The DM group was fed a HFD for 12 weeks, and the control group was fed a NCD for 12 weeks. $^{\#}P < 0.01$ vs. WT + GFP. $^{*}P < 0.01$ vs.

KO + GFP. One-way ANOVA followed by Tukey's post-test for multiple comparisons was used for groups of three or more.

Abbreviations: SBP, systolic blood pressure. DBP, diastolic blood pressure. FBG, fasting blood glucose. HbA1C, hemoglobin A1c. TC, total cholesterol. TG, triglycerides. FFA, free fatty acid. LDL-C, low-density lipoprotein-cholesterol. HDL-C, high-density lipoprotein-cholesterol. MYDGF, myeloid-derived growth factor.

Table S5 A list of RT-PCR primers used in this study.

Primer name	Forward primer (5' to 3')	Reverse primer (5' to 3')
Mydgf	CTGAAGTGAGTCCGGGAGC	AGTGGGAAAGGGACGGGTG
Srebp1c	CTGGGGGTGAGACAGGGGAC	GATGGTGGAGGGGACAAGGG
Fasn	CTGCGGAAACTTCAGGAAATG	GGTTCGGAATGCTATCCAGG
Acc1	GGCCAGTGCTATGCTGAGAT	AGGGTCAAGTGCTGCTCCA
Scd1	GAGGCCTGTACGGGATCATA	TGAGAGAAGAAGAAGCCACGG
Cpt1a	TGGGCTACTCAGAGGATGG	AAGGTGTCAAATGGGAAGG
Acox1	GCCAAGGCGACCTGAGTGAGC	ACCGCAAGCCATCCGACATTC
Pparg	GCAGCTACTGCATGTGATCAAGA	GTCAGCGGGTGGGACTTTC
Tnf-α	AGCCCCCAGTCTGTATCCTT	CTCCCTTTGCAGAACTCAGG
Il-1β	CCGTGGACCTTCCAGGATGA	GGGAACGTCACACACCAGCA
Il-6	ACTCCAGAAGACCAGAGGAAAT	CCAGAGATACAAAGAAATGATGG
Gapdh	TCAACAGCAACTCCCACTCTTCCA	TTGTCATTGAGAGCAATG CCAGCC

Abbreviations: *Mydgf*, myeloid-derived growth factor. *Srebp1c*, sterol regulatory element-binding protein 1c. *Fasn*, fatty acid synthase. *Acc1*, acetyl-CoA carboxylase 1. *Scd1*, stearoyl-CoA desaturase 1. Cpt1a, carnitine palmitoyltransferase 1a. *Acox1*, acyl-coenzyme A oxidase 1. *Pparg*, peroxisome proliferator-activated receptor gamma. *Tnf-α*, tumor necrosis factor-α. *Il-1β*, interleukin-1β. *Il-6*, interleukin-6. *Gapdh*, glyceraldehyde 3-phosphate dehydrogenase.

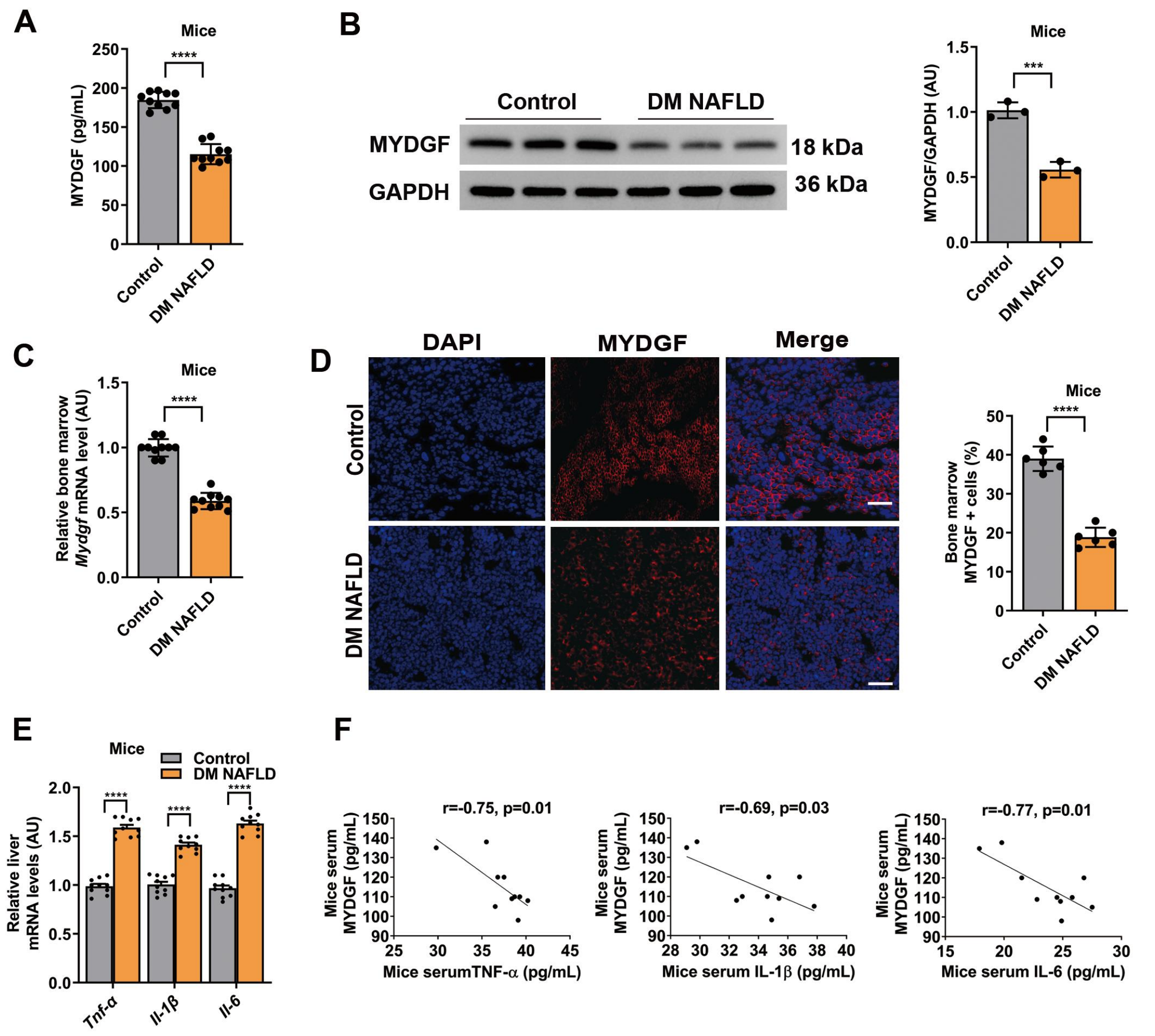

Figure S1 Reduced MYDGF levels and enhanced inflammation in NAFLD mice. **(A)** The serum MYDGF levels in DM NAFLD and control mice. **(B)** Mice bone marrow MYDGF protein levels. **(C)** Mice bone marrow *Mydgf* mRNA levels. **(D)** Expression of MYDGF (red) in mice bone marrow. Nuclei are shown in blue. White bar = 20 μm. **(E)** TNF-α, IL-1β, and IL-6 mRNA levels in the liver. **(F)** Correlation between serum MYDGF levels and serum TNF-α, IL-1β, and IL-6 levels in DM NAFLD mice. Data are presented as the mean ± SEM. $^{***}P < 0.001$, $^{****}P < 0.0001$. Significant differences were determined by Student's *t*-test or one-way ANOVA followed by Tukey's post-test. Abbreviations: DM, diabetes mellitus. TNF-α, tumor necrosis factor-α. IL-1β, interleukin-1β. IL-6, interleukin-6.

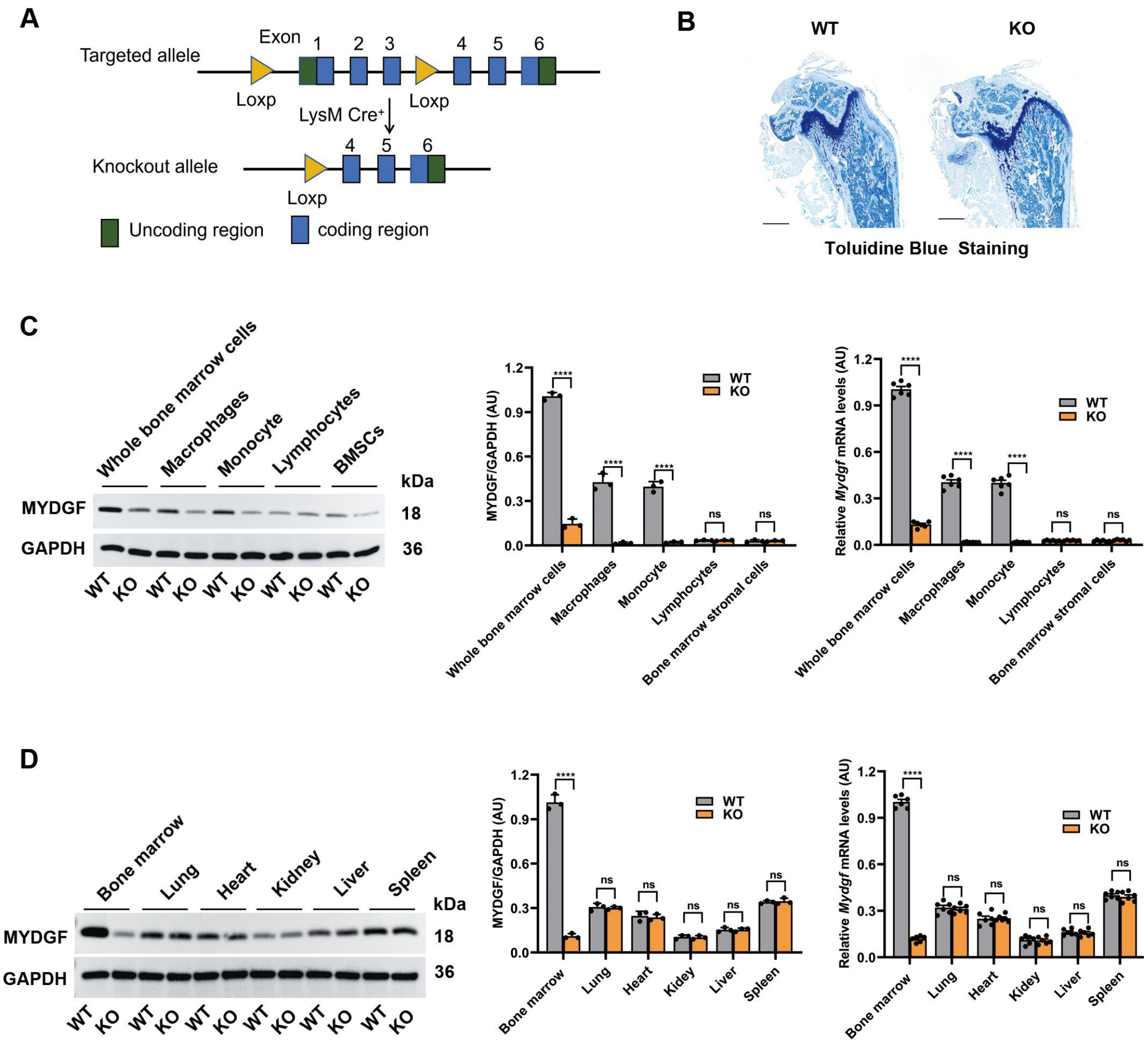

Figure S2 Bone marrow integrity and the expression of MYDGF in WT and KO mice. (A) The strategy for myeloid cell-specific MYDGF knockout mice. **(B)** Representative images showing toluidine blue staining of femur sections in WT and KO mice. Black bar = 500 μm. **(C)** Mice MYDGF protein levels and *Mydgf* mRNA levels in whole bone marrow cells, hematopoietic cell subtypes, and bone marrow stromal cells of WT and KO mice. **(D)** Mice MYDGF protein levels and *Mydgf* mRNA levels in other tissues of WT and KO mice. $^{****}P < 0.0001$. Significant differences were determined by Student's *t*-test or one-way ANOVA followed by Tukey's post-test. Abbreviations: WT, wild type. KO, *Mydgf* knock out. MYDGF, myeloid-derived growth factor. GAPDH, glyceraldehyde-3-phosphate dehydrogenase.

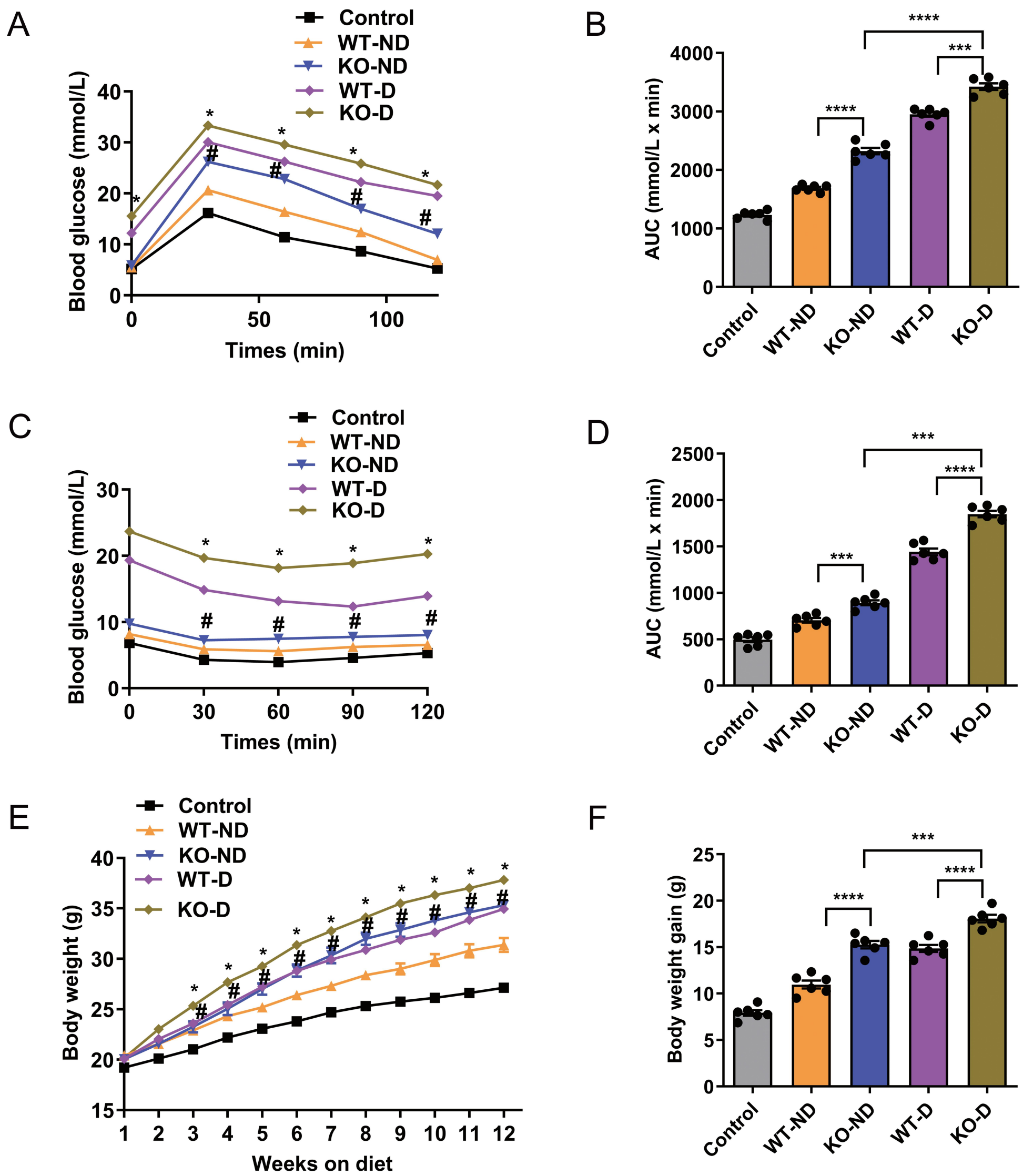

Figure S3 Myeloid cell-specific MYDGF deficiency deteriorated the metabolic profiles of DM NAFLD mice. HFD/STZ-induced DM models in WT and KO mice, and fed a HFD for 12 weeks. The control group indicates WT mice fed an NCD. **(A) The** results of the GTT at the end of the experiment. $^{\#}P < 0.001$ vs. WT-ND group. $^{*}P < 0.001$ vs. WT-D group. **(B)** The area under the curve (AUC) from the results of the GTT. $^{***}P < 0.001$, $^{****}P < 0.0001$. **(C)** The results of ITT at the end of the experiment. $^{\#}P < 0.001$ vs. WT-ND group. $^{*}P < 0.001$ vs. WT-D group. **(D)** The AUC from the results of the ITT. $^{***}P < 0.001$, $^{****}P < 0.0001$. **(E)** Body weight. $^{\#}P < 0.001$ vs.

WT-ND group. *$P < 0.001$ vs. WT-D group. **(F)** Body weight gain. ***$P < 0.001$, ****$P < 0.0001$. Data are presented as the mean ± SEM. Significant differences were determined by Student's *t*-test or one-way ANOVA followed by Tukey's post-test. Abbreviations: WT, wild type. KO, *Mydgf* knock out. DM, diabetes mellitus. ND, non-diabetes mellitus. HFD, high-fat diet. STZ, streptozotocin. NCD, normal chow diet. GTT, glucose tolerance test.

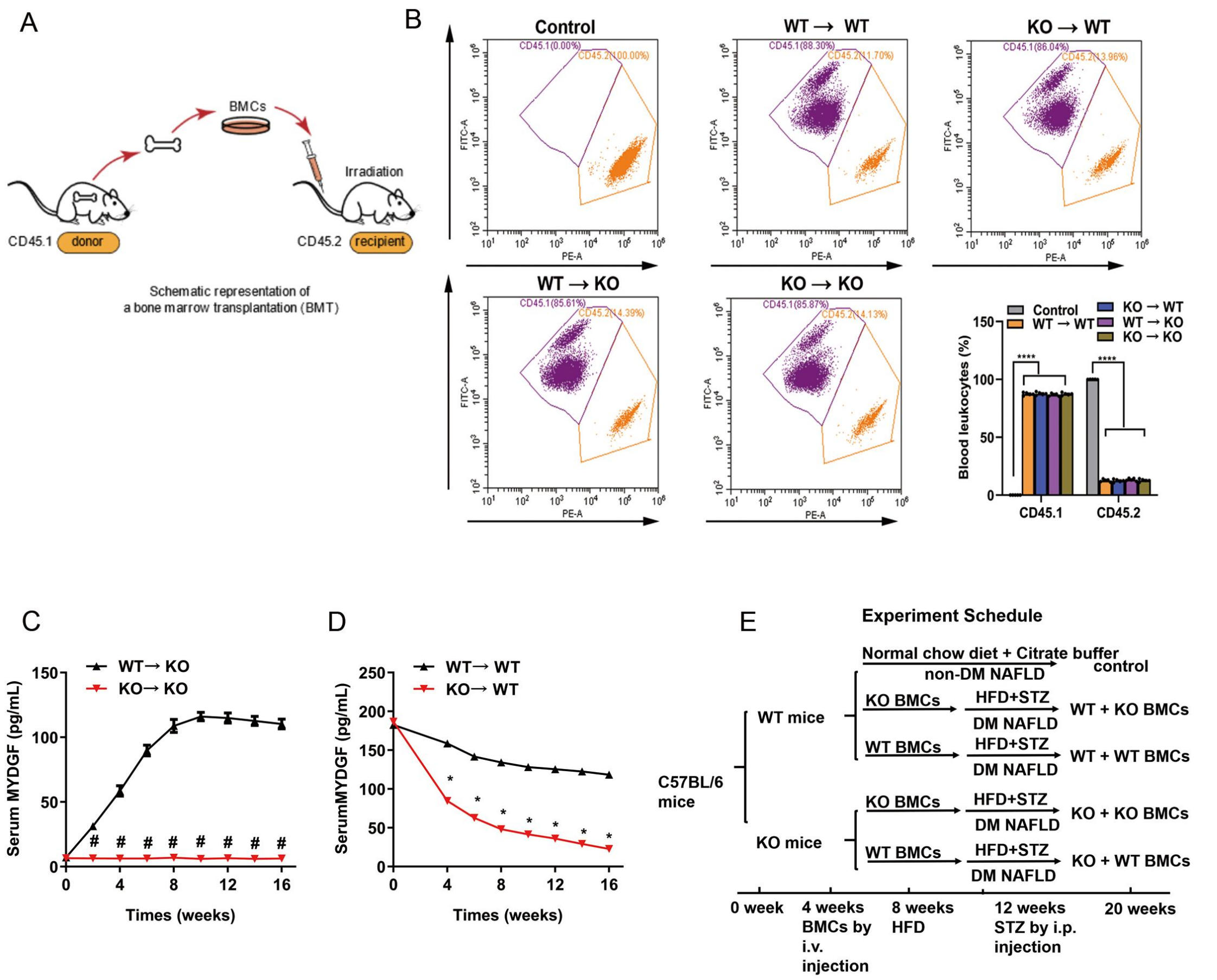

Figure S4 The expression of MYDGF after BMT in mice. BMT was performed in WT and KO mice aged 4 weeks, HFD/STZ-induced DM models in WT and KO mice, and fed a HFD for 12 weeks. The control group indicates WT mice fed with NCD. **(A)** Schematic representation of BMT. **(B)** Blood leukocytes were analyzed for CD45.1 and CD45.2 expression by flow cytometry after BMT. **(C)** Serum levels of MYDGF in KO mice at different time points after BMT. **(D)** Serum levels of MYDGF in WT mice at different time points after BMT. **(E)** The experimental schedule used to assess the effects of BMT on NAFLD. Data are presented as mean ± SEM. $^{\#}P < 0.001$ vs. WT→KO group. $^{*}P < 0.001$ vs. WT→WT group. Data are presented as mean ± SEM. Significant differences were determined by Student's *t*-test or one-way ANOVA

followed by Tukey's post-test. Abbreviations: BMT, bone marrow transplantation. HFD, high-fat diet. STZ, streptozotocin. DM, diabetes mellitus. NCD, normal chow diet. WT, wild type. KO, *Mydgf* knock out. NAFLD, non-alcoholic fatty liver disease. MYDGF, myeloid-derived growth factor. BMCs, bone marrow cells.

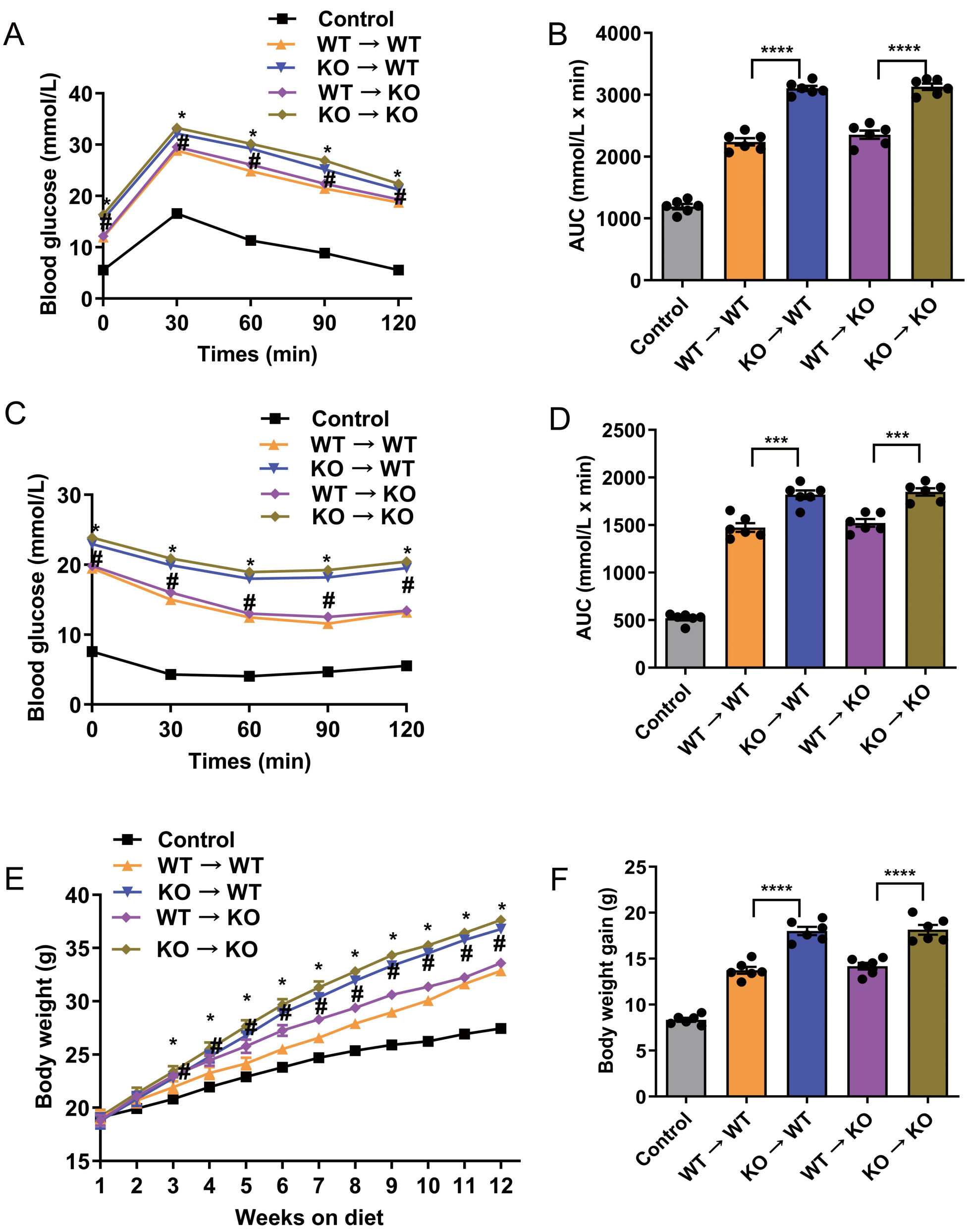

Figure S5 BMT improved metabolic profiles in DM NAFLD mice. (A) The results of the GTT at the end of the experiment. $^{\#}P < 0.001$ vs. WT → WT group. $^{*}P < 0.001$ vs. WT → KO group. **(B)** The AUC from the results of the GTT. $^{****}P < 0.0001$. **(C)** The results of ITT at the end of the experiment. $^{\#}P < 0.001$ vs. WT → WT group. $^{*}P < 0.001$ vs. WT → KO group. **(D)** The AUC from the results of the ITT. $^{***}P < 0.001$. **(E)** Body weight. $^{\#}P < 0.001$ vs. WT → WT group. $^{*}P < 0.001$ vs. WT → KO group. **(F)** Body weight gain. $^{****}P < 0.0001$. Data are presented as the mean ±

SEM. Significant differences were determined by Student's *t*-test or one-way ANOVA followed by Tukey's post-test. Abbreviations: BMT, bone marrow transplantation. HFD, high-fat diet. STZ, streptozotocin. DM, diabetes mellitus. WT, wild type. KO, *Mydgf* knock out. NAFLD, non-alcoholic fatty liver disease.

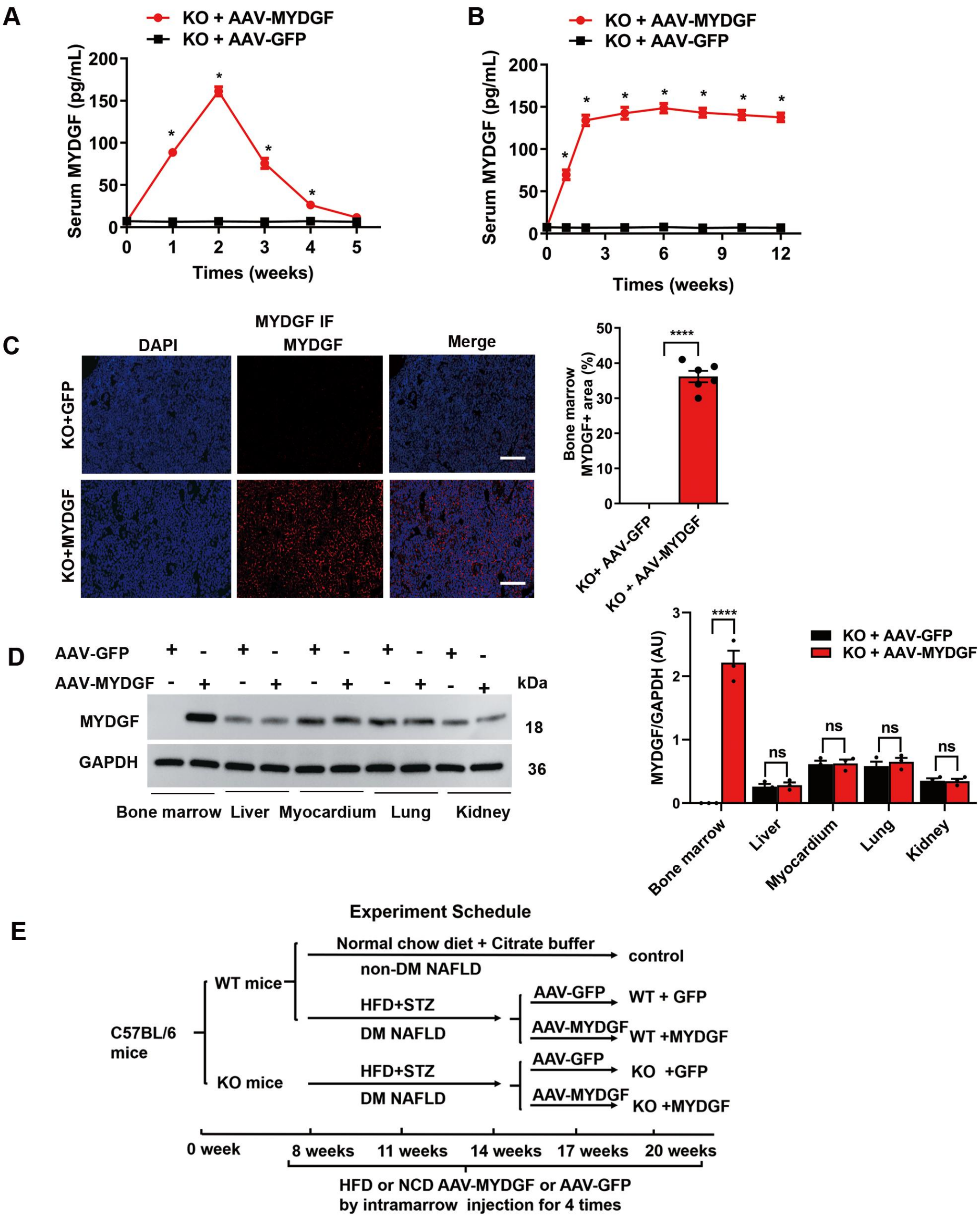

Figure S6 The expression of MYDGF after intramarrow injection of AAV-MYDGF in mice. (A-D) KO mice aged 4 weeks were selected, and intramarrow AAV-MYDGF injection was performed. All mice were fed with NCD. **(A)** Serum levels of MYDGF at different time points after a single injection of AAV. $^{*}P < 0.001$ vs. KO + AAV-GFP group. **(B)** Serum levels of MYDGF at different time points after injection of AAV every 3 weeks (8 weeks, 11 weeks, 14 weeks, 17

weeks). $^{*}P < 0.001$ vs. KO + AAV-GFP group. **(C)** The expression of MYDGF (red) in bone marrow at 12 weeks. White scale bar = 20 μm. $^{****}P < 0.0001$. **(D)** MYDGF protein levels in bone marrow, liver, myocardium, lung, and kidney in mice at 12 weeks. $^{****}P < 0.001$. **(E)** The experimental schedule used to assess the effects of MYDGF expression in bone marrow on DM NAFLD after intramarrow AAV injection. AAV injection was performed in WT and KO mice aged 4 weeks, HFD/STZ-induced DM models in WT and KO mice, and fed a HFD for 12 weeks. The control group indicates WT mice fed with NCD. Data are presented as the mean ± SEM. Significant differences were determined by Student's *t*-test or one-way ANOVA followed by Tukey's post-test. Abbreviations: HFD, high-fat diet. STZ, streptozotocin. DM, diabetes mellitus. NCD, normal chow diet. NAFLD, non-alcoholic fatty liver disease. ND, non-diabetes mellitus. WT, wild type. KO, *Mydgf* knock out. AAV, adeno-associated virus. GFP, green fluorescent protein. MYDGF, myeloid-derived growth factor. GAPDH, glyceraldehyde-3-phosphate dehydrogenase.

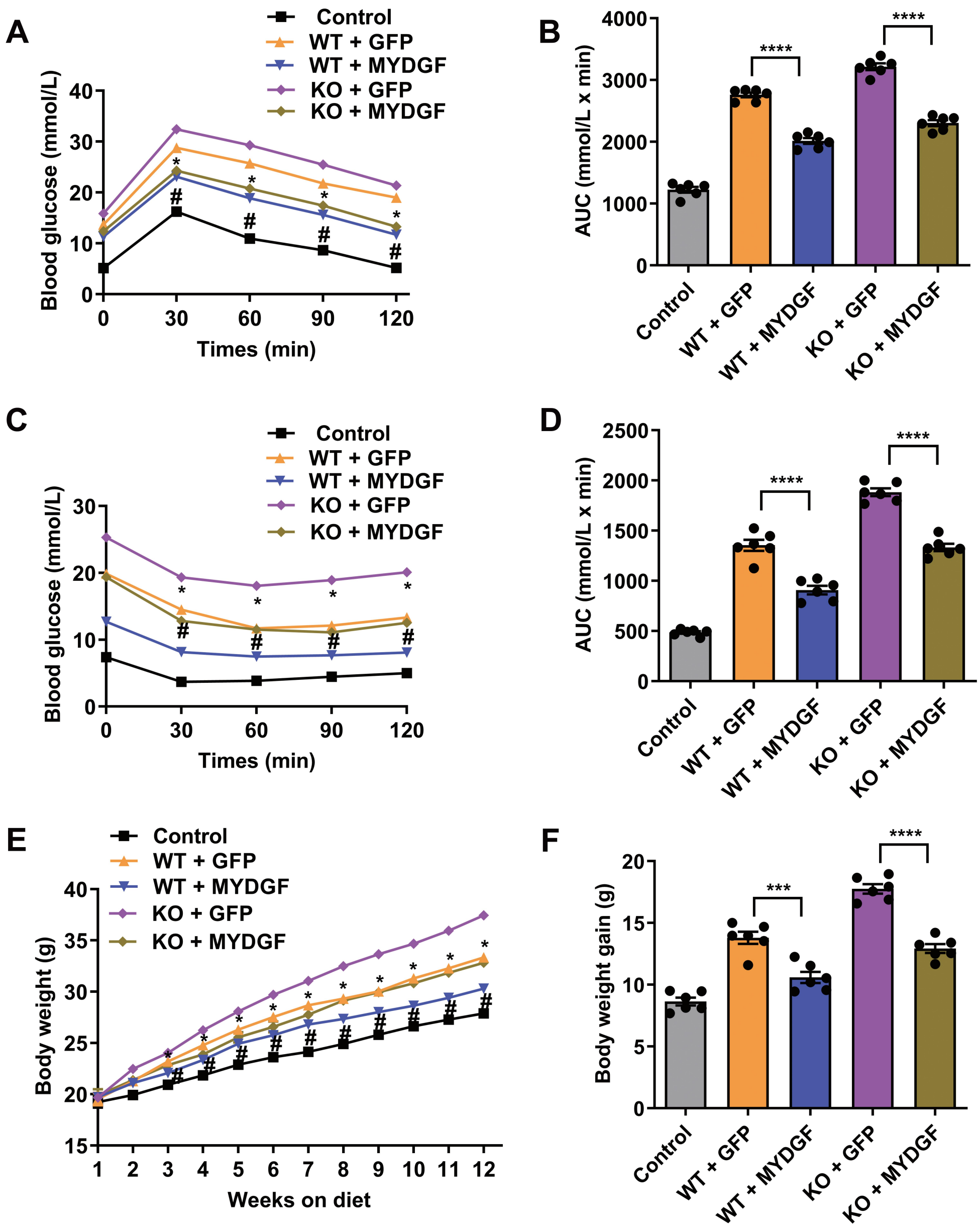

Figure S7 Bone marrow-specific overexpression of MYDGF improved metabolic profiles in DM NAFLD mice. KO and WT mice aged 4–6 weeks were selected, and intramarrow AAV-MYDGF injection was done as indicated in Supplementary Figure 6E. HFD/STZ-induced DM models in WT and KO mice were fed an HFD for 12 weeks. The control group was fed an NCD. **(A)** The results of the GTT at the end of the experiment. $^{\#}P < 0.001$ vs. WT-GFP group. $^{*}P < 0.001$ vs. KO-GFP group. **(B)** The AUC from the results of the GTT. $^{****}P < 0.0001$. **(C)** The results of ITT at the end of

the experiment. $^{\#}P < 0.001$ vs. WT-GFP group. $^{*}P < 0.001$ vs. KO-GFP group. **(D)** The AUC from the results of the ITT. $^{****}P < 0.0001$. **(E)** Body weight. $^{\#}P < 0.001$ vs. WT-GFP group. $^{*}P < 0.001$ vs. KO-GFP group. **(F)** Body weight gain. $^{***}P < 0.001$, $^{****}P < 0.0001$. Data are presented as the mean ± SEM. Significant differences were determined by Student's *t*-test or one-way ANOVA followed by Tukey's post-test. Abbreviations: HFD, high-fat diet. STZ, streptozotocin. DM, diabetes mellitus. NCD, normal chow diet. NAFLD, non-alcoholic fatty liver disease. WT, wild type. KO, *Mydgf* knock out. AAV, adeno-associated virus. GFP, green fluorescent protein. MYDGF, myeloid-derived growth factor. GTT, glucose tolerance test.

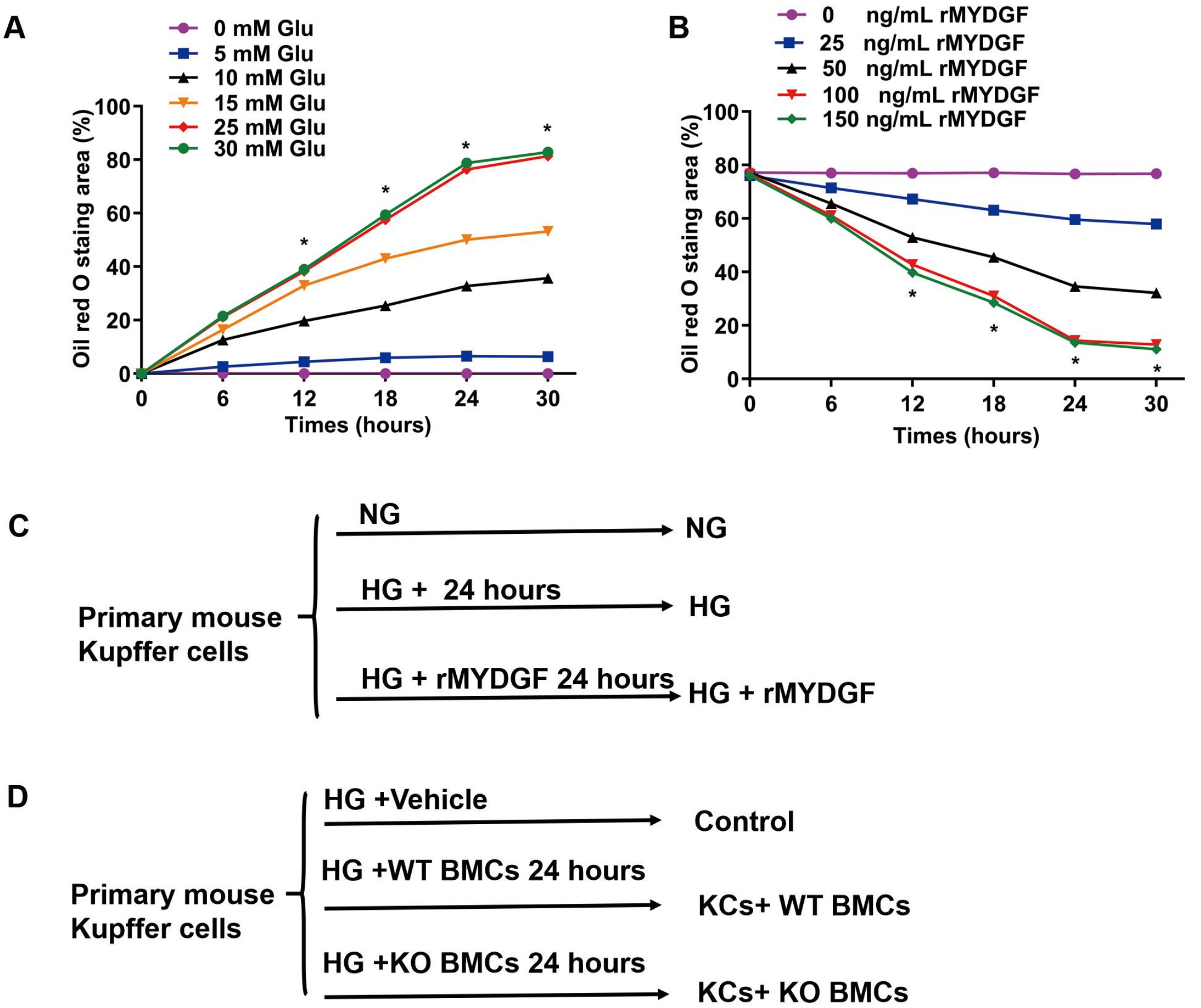

Figure S8 Identification of the optimum conditions for the incubation of KCs with HG and rMYDGF. **(A)** Time- and dose-dependent glucose-induced change in fat deposition determined by oil red O staining. **(B)** Time- and dose-dependent HG-induced changes in fat deposition following rMYDGF incubation were determined by Oil Red O staining. **(C-D)** The experimental schedule used to assess the effects of MYDGF on HG-induced KCs treated with rMYDGF (**C**) or cocultured with BMCs (**D**). Data are presented as the mean ± SEM. $^{*}P$ <0.001 vs. control group. Significant differences were determined by Student's *t*-test or one-way ANOVA followed by Tukey's post-test. Abbreviations: rMYDGF, recombinant myeloid-derived growth factor. NG, normal glucose. HG, high glucose. KCs, Kupffer cells. WT, wild type. KO, *Mydgf* knock out. Glu, glucose. BMCs, bone marrow cells.

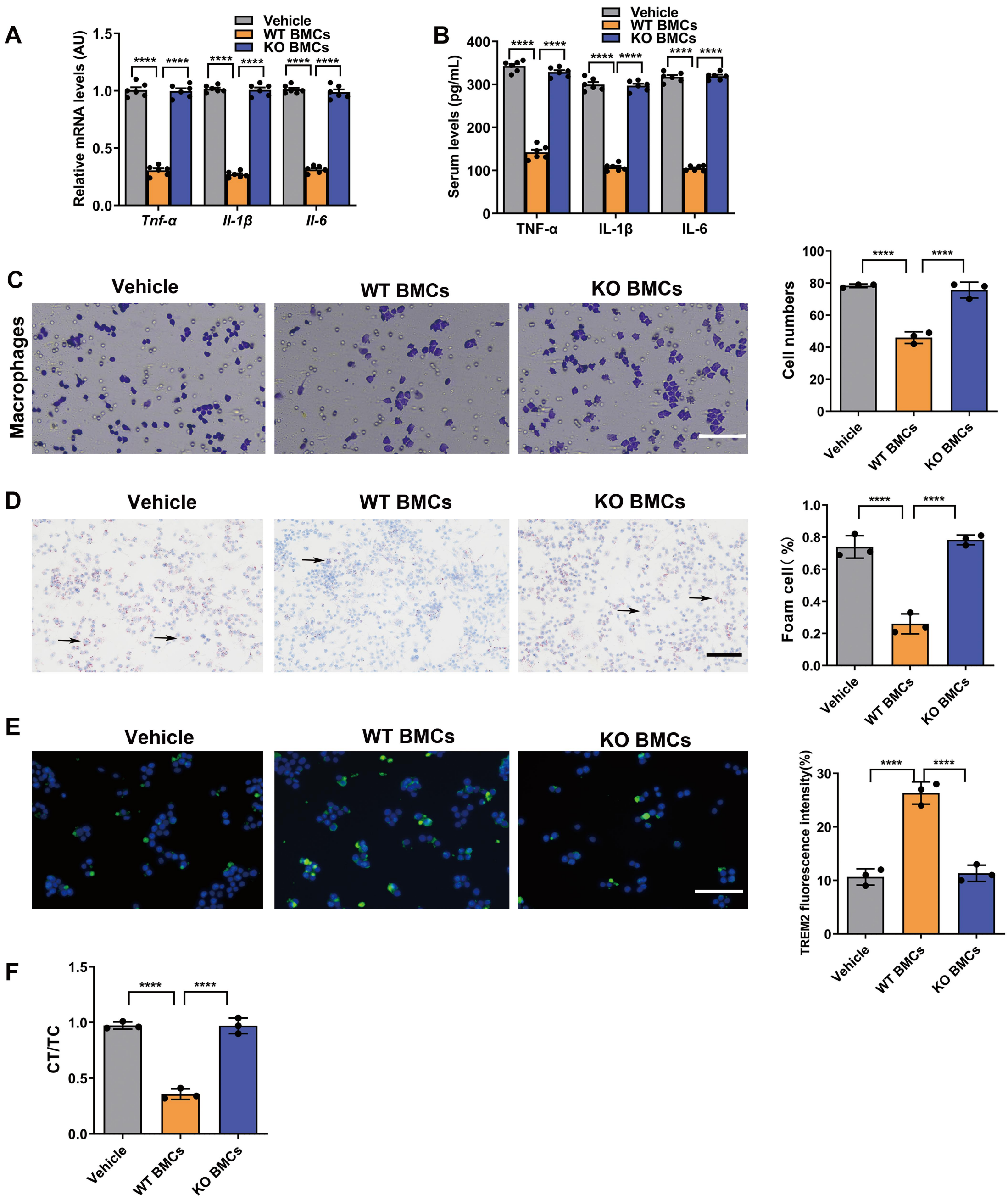

Figure S9 MYDGF improved inflammation, reduced macrophage foaming, reduced cholesterol content in macrophages, and enhanced macrophage efferocytosis in co-culture experiments. KCs were treated as described in Figure S8D. **(A)** The mRNA levels of *Tnf-α, Il-1β,* and *Il-6* in macrophages cocultured with BMCs. **(B)** The levels of inflammatory factors in supernatants of macrophages cocultured with BMCs. **(C)** Representative migration images of macrophages in

macrophages cocultured with BMCs. Scale bar, 100 μm. **(D)** Representative images showing Oil Red O staining in macrophages cocultured with BMCs. Black arrows indicate foam cells. **(E)** Representative images showing TREM2 immunofluorescence staining in macrophages co-cultured with BMCs. **(F)** The levels of cholesterol ester/total cholesterol in macrophages co-cultured with BMCs. Data are presented as the mean ± SEM. $^{**}P < 0.01$, $^{***}P < 0.001$, $^{****}P < 0.0001$. Significant differences were determined by Student's *t*-test or one-way ANOVA followed by Tukey's post-test. Abbreviations: KCs, Kupffer cells. WT, wild type. KO, *Mydgf* knock out. Vehicle, HG. HG, high glucose. BMCs, bone marrow cells. TNF-α, tumor necrosis factor-α. IL-1β, interleukin-1β. IL-6, interleukin-6. CE, cholesterol ester. TC, total cholesterol.

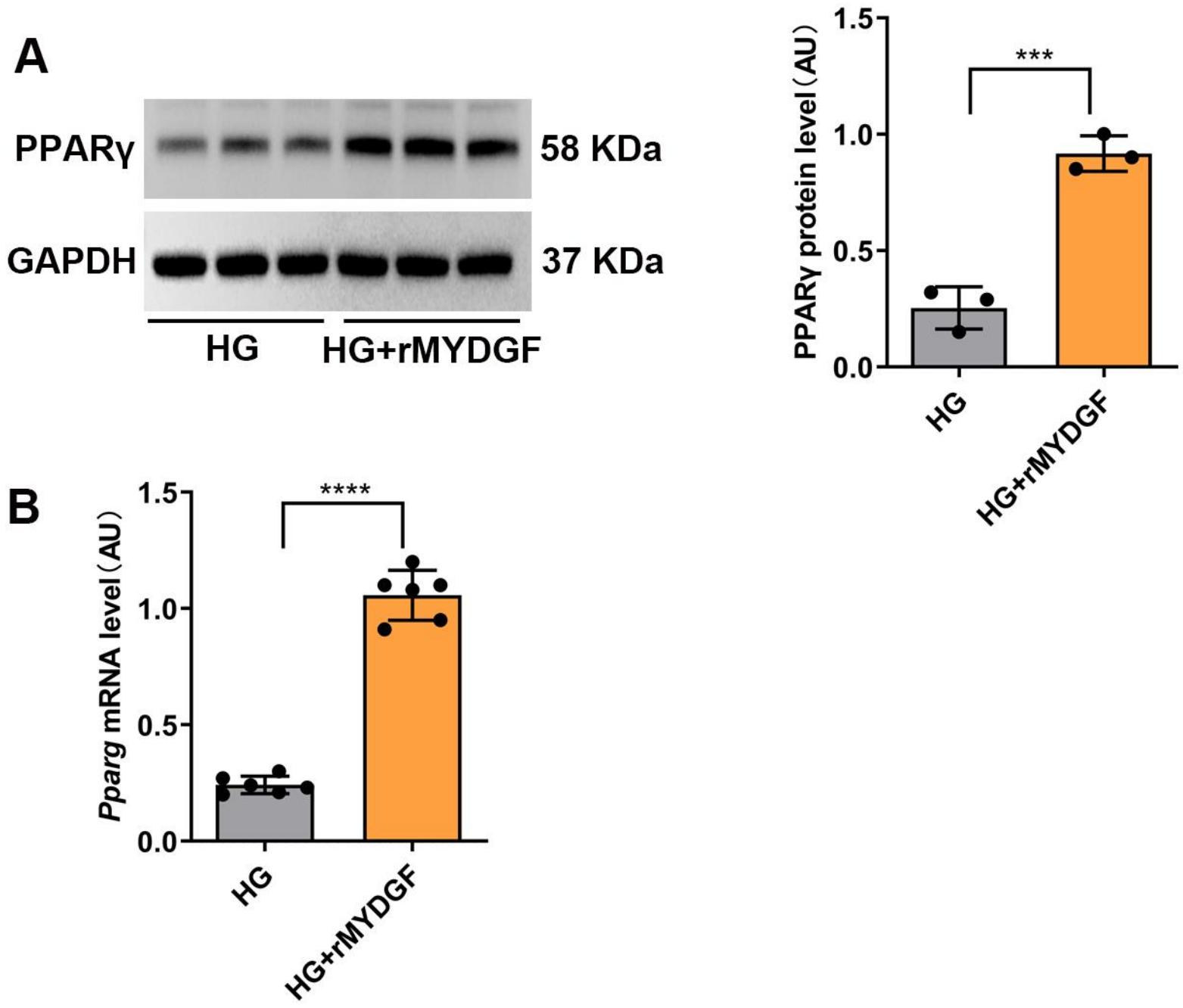

Figure S10 Expression of PPARγ protein and mRNA in two groups of macrophages. (A, B) PPARγ protein and mRNA expression in two groups of macrophages. Data are presented as the mean ± SEM. $^{***}P < 0.001$, $^{****}P < 0.0001$. Significant differences were determined by Student's *t*-test. Abbreviations: rMYDGF, recombinant myeloid-derived growth factor. HG, high glucose. KCs, Kupffer cells. PPARγ, peroxisome proliferator-activated receptor gamma. GAPDH, glyceraldehyde-3-phosphate dehydrogenase.

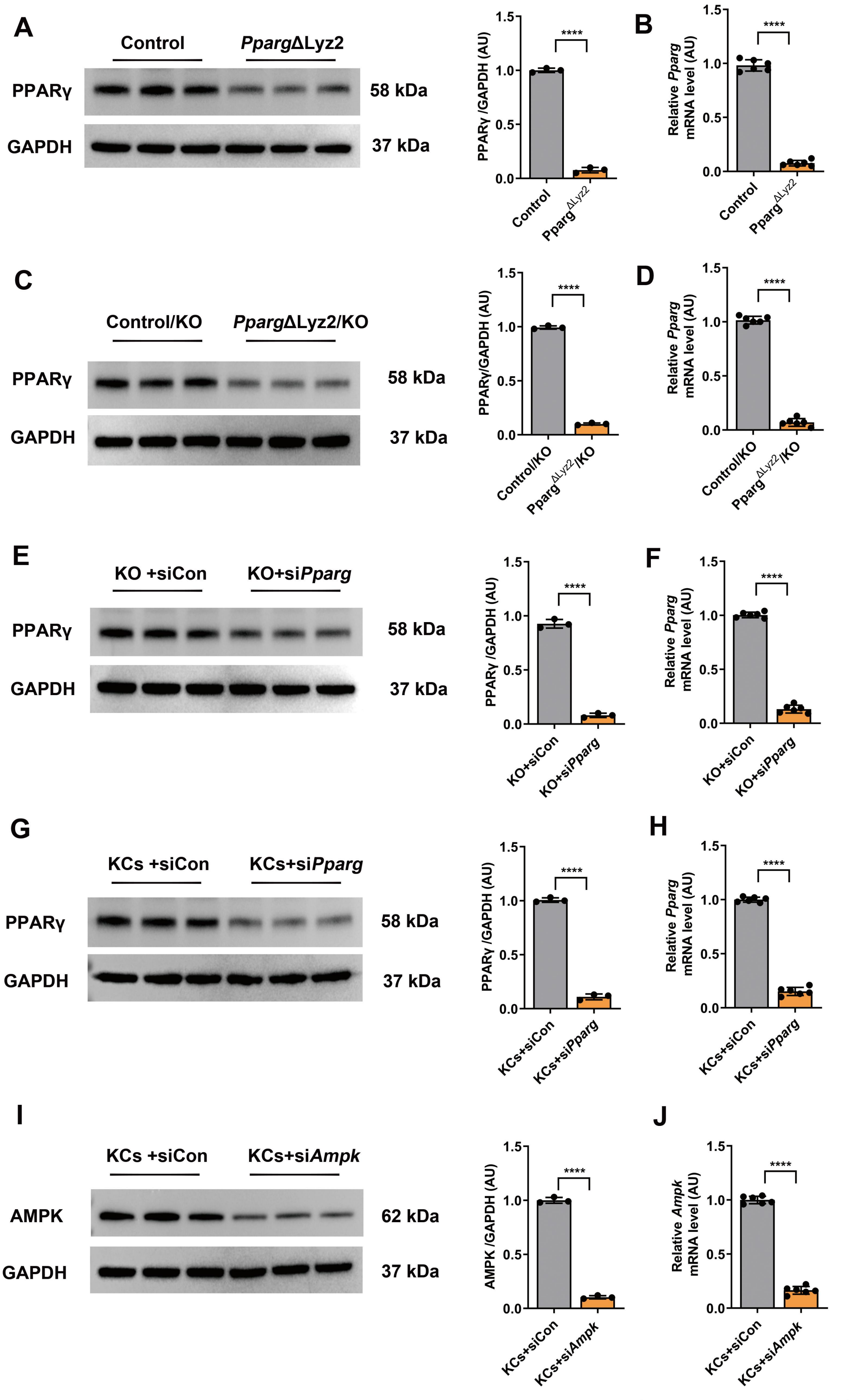

Figure S11 The confirmation of *Pparg* knockdown in *Pparg*$^{\Delta Lyz2}$ mice and *Pparg*$^{\Delta Lyz2}$/KO mice or silencing in liver *in vitro*, as well as the confirmation of

***Pparg* or *Ampk* knockdown after siCon and si*Pparg* or si*Ampk* treatment in KCs.**

(A) The PPARγ protein expression in BMDMs from mice. **(B)** The *Pparg* mRNA expression in BMDMs from mice. **(C)** The PPARγ protein expression in BMDMs from mice. **(D)** The *Pparg* mRNA expression in BMDMs from mice. **(E)** Liver PPARγ protein levels in mice after a single injection of AAV siRNA. **(F)** Liver *Pparg* mRNA levels in mice after a single injection of AAV siRNA. (**G–H**) KCs were transfected with si*Pparg* and siCon. After 12 hours, another treatment with 25 mM HG or 100 ng/mL rMYDGF for 24 hours. **G** PPARγ protein levels in KCs from WT mice after transfection with siRNA for *Pparg*. **H** *Pparg* mRNA levels in KCs from WT mice after transfection with siRNA for *Pparg*. (**I-J**) KCs were transfected with si*Ampk* and siCon. After 12 hours, another treatment with 25 mM HG or 100 ng/mL rMYDGF for 24 hours. **(I)** AMPK protein levels in KCs from WT mice after transfection with siRNA for *Ampk*. (**J**)*Ampk* mRNA levels in KCs from WT mice after transfection with siRNA for *Ampk*. Data are presented as the mean ± SEM. $^{****}P < 0.0001$. Significant differences were determined by Student's *t*-test. Abbreviations: rMYDGF, recombinant myeloid-derived growth factor. HG, high glucose. KCs, Kupffer cells. WT, wild type. KO, *Mydgf* knock out. BMCs, bone marrow cells. PPARγ, peroxisome proliferator-activated receptor gamma. AMPK, AMP-activated protein kinase. GAPDH, glyceraldehyde-3-phosphate dehydrogenase.

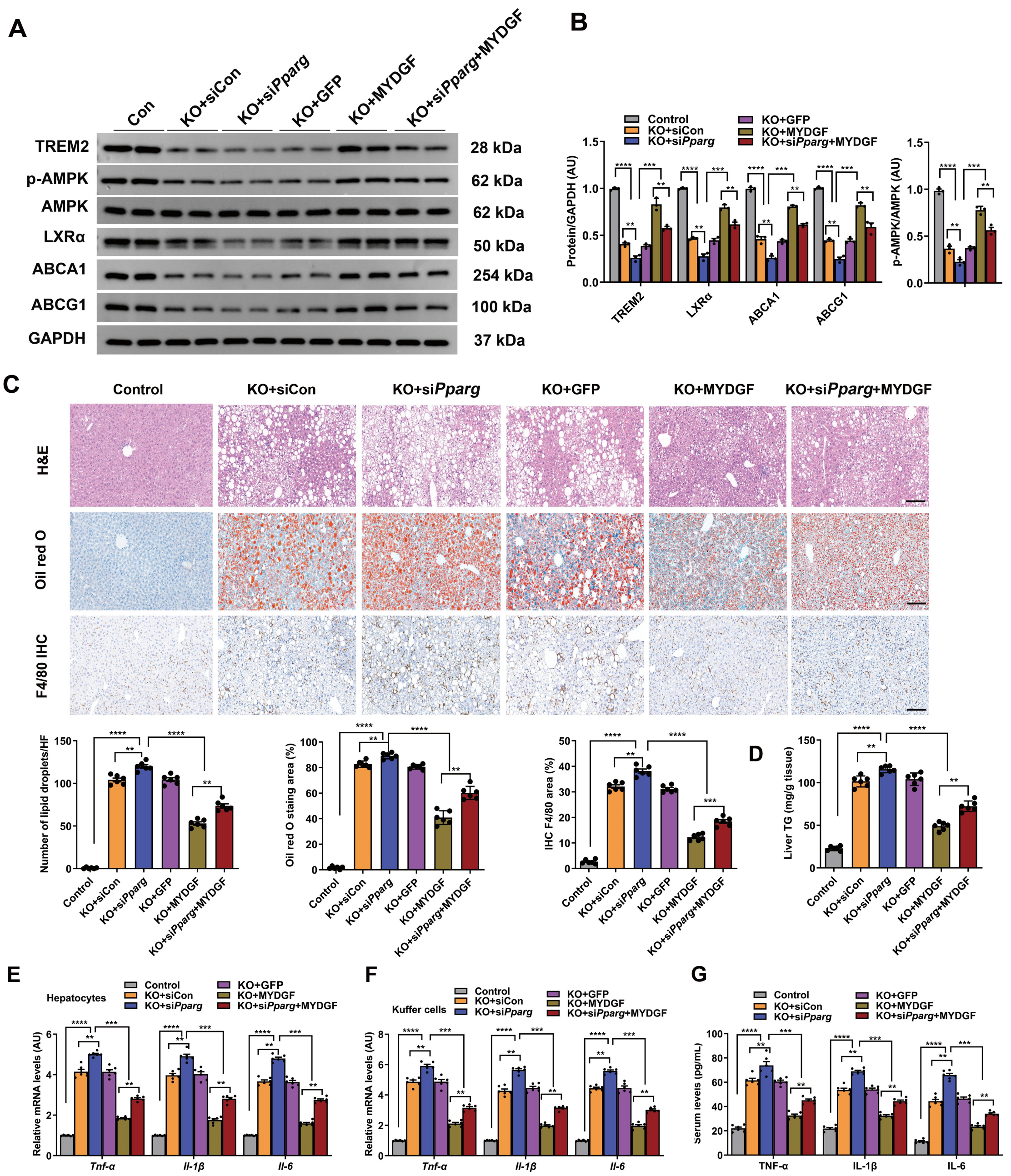

Figure S12 AMPK/PPARγ/LXRα signaling pathway is involved in the beneficial effects of MYDGF in DM NAFLD *in vivo*. KO and siPPARγ/KO received intramarrow injection of AAV-GFP or AAV-MYDGF every 3 weeks for 12 weeks, underwent DM modeling, and were fed a HFD for 12 weeks. **(A, B)** AMPK/PPARγ/LXRα pathway proteins in the liver macrophages of mice. **(C)** Representative images showing HE staining, oil red O staining, and F4/80 staining of

liver sections. Black scale bar: 50 μm. **(D)** Hepatic TG content. **(E)** Levels of inflammatory factors in isolated hepatocytes. **(F)** Levels of inflammatory factors in KCs. **(G)** Levels of inflammatory factors in serum. Data are presented as the mean ± SEM. $^{**}P < 0.01$, $^{***}P < 0.001$, $^{****}P < 0.0001$. Significant differences were determined by Student's *t*-test or one-way ANOVA followed by Tukey's post-test. Abbreviations: KO, *Mydgf* knock out. DKO, *Pparg* and *Mydgf* double knock out. DM, diabetes mellitus. NAFLD, non-alcoholic fatty liver disease. MYDGF, myeloid-derived growth factor. KCs, Kupffer cells. TREM2, triggering receptor expressed on myeloid cells. AMPK, AMP-activated protein kinase. PPARγ, peroxisome proliferator-activated receptor gamma. LXRα, liver X receptor α. ABCA1, ATP-binding cassette transporter A1. ABCG1, ATP-binding cassette transporter G1. GAPDH, glyceraldehyde-3-phosphate dehydrogenase.
